# Multi-omics and single cell characterization of cancer immunosenescence landscape

**DOI:** 10.1038/s41597-024-03562-z

**Published:** 2024-07-07

**Authors:** Qiuxia Wei, Ruizhi Chen, Xue He, Yanan Qu, Changjian Yan, Xiaoni Liu, Jing Liu, Jiahao Luo, Zining Yu, Wenping Hu, Liqun Wang, Xiaoya Lin, Chaoling Wu, Jinyuan Xiao, Haibo Zhou, Jing Wang, Mingxia Zhu, Ping Yang, Yingtong Chen, Qilong Tan, Xiaoliang Yuan, Hongmei Jing, Weilong Zhang

**Affiliations:** 1 Department of Hematology, Lymphoma Research Center, Peking University Third Hospital, Beijing, 100191 China; 2 Gannan Medical University, Ganzhou, 341000 China; 3 Suichang County People’s Hospital, Lishui, 323000 China; 4 Department of Pathology, Beijing Tiantan Hospital, Capital Medical University, Beijing, 100070 China; 5 Peking University Research Center on Aging, Department of Biochemistry and Molecular Biology, School of Basic Medical Sciences, Peking University Health Science Center, 100191 Beijing, China; 6 The Second Affiliated Hospital of Fujian Medical University, Quanzhou, 362000 China; 7 Department of Respiratory Medicine, The First Affiliated Hospital of Gannan Medical University, Ganzhou, 341000 China; 8 Department of Clinical Laboratory, Shangrao Municipal Hospital, Jiangxi, 334000 China; 9 Department of Radiation Oncology, Harbin Medical University Cancer Hospital, Harbin, 150000 China; 10 Department of Respiratory medicine, Affiliated Hospital of Jiujiang University, Jiujiang, 332000 China; 11 Department of Epidemiology & Health Statistics, School of Public Health, School of Medicine, Zhejiang University, Hangzhou, 310058 China; 12 School of Public Health, School of Medicine, Zhejiang University, Hangzhou, 310058 China

**Keywords:** Cancer microenvironment, Risk factors

## Abstract

Cellular senescence (CS) is closely related to tumor progression. However, the studies about CS genes across human cancers have not explored the relationship between cancer senescence signature and telomere length. Additionally, single-cell analyses have not revealed the evolutionary trends of malignant cells and immune cells at the CS level. We defined a CS-associated signature, called “senescence signature”, and found that patients with higher senescence signature had worse prognosis. Higher senescence signature was related to older age, higher genomic instability, longer telomeres, increased lymphocytic infiltration, higher pro-tumor immune infiltrates (Treg cells and MDSCs), and could predict responses to immune checkpoint inhibitor therapy. Single-cell analysis further reveals malignant cells and immune cells share a consistent evolutionary trend at the CS level. MAPK signaling pathway and apoptotic processes may play a key role in CS, and senescence signature may effectively predict sensitivity of MEK1/2 inhibitors, ERK1/2 inhibitors and BCL-2 family inhibitors. We also developed a new CS prediction model of cancer survival and established a portal website to apply this model (https://bio-pub.shinyapps.io/cs_nomo/).

## Introduction

Aging is one of the important causes of cancer, with epidemiological investigations indicating that the cancer incidence increases with age^1^. Cellular senescence (CS) contributes to aging in humans^2^. CS is considered to be a state of cell proliferation arrest due to the cell cycle being halted^3^. Both cancer and CS result from the time-dependent accumulation of cellular damage, and many studies have shown a substantial overlap between the hallmarks of CS and cancer^4^. These hallmarks include epigenetic changes, altered signaling pathways, chromosomal instability, changes in protein homeostasis, damage to telomeres, mitochondrial dysfunction, and more^4,5^. In particular, the discovery of the senescence-associated secretory phenotype (SASP)^6^ suggests that senescent cells can promote tumor progression and metastasis by influencing the tumor microenvironment (TME)^7^.

Antitumor acquired immune programs can be tampered with by factors secreted by senescent cells in the TME, which regulate the malignant behavior of tumor by adjusting the components and functions of the innate immune system^8^. Extensive microarray analyses have manifested that the secretory expression profile of senescent cells, called SASP, includes growth factors, inflammatory cytokines, chemokines, and proteases. The SASP inhibits antitumor immune mechanisms by inducing immunosuppressive immune infiltrates^9^, promotes tumor malignant behavior by secreting growth factors, enhancing angiogenesis and remodeling of the extracellular matrix (ECM)^6^, and possesses high resistance to therapy^10–12^. Interleukin-6 (IL-6) is the most prominent cytokine of the SASP and senescent stromal cells have been proven to promote immunosuppressive immune infiltrates, such as myeloid-derived suppressor cells (MDSCs) and regulatory T (Treg) cells, in mouse experiments through the secretion of IL-6^9^. Meanwhile, senescent cells can autocrine type I interferon (IFN-1) to maintain the SASP and sustain immunosuppression^13^.

The CS has a significant impact on TME, meaning great potential for predicting immune therapy responses. However, there are few studies on the response to targeted therapy or immunotherapy in cellular characteristics of senescent cells. Most studies focus on age prediction of immunotherapy response, which remains controversial. A thorough review of published datasets has revealed that currently available immune checkpoint inhibitors are very effective for the elderly^14^. In melanoma patients, the likelihood of response to anti-PD1 therapy increases with age, a finding that has been replicated in young and aged melanoma mouse models^15^. However, in breast cancer, young mouse models show a significantly better response to anti-PDL1 or anti-CTLA4 treatment compared to aged mouse models^16^. Consequently, it is necessary to explore the heterogeneity of CS in various tumor tissues and unify quantitative standards for CS to predict immune therapy responses.

The CS can be divided into two types based on their mechanisms. The first type, known as replicative senescence, involves the persistent inhibition of cell proliferation discovered by Hayflick, which relies on the continuous shortening of telomeres^3,17^. This type is mediated by the p14-p53-p21-Rb pathway^18,19^. The second type, termed premature senescence^20^, is a stress-induced accelerated senescence response independent of telomere shortening and is mediated by the ATM-p53-p21-Rb and p16-Rb pathways^21–23^. Although the two types of CS are distinguished by whether telomere shortening occurs, telomere damage is present in both. Half of the continuous DNA damages are pinned to telomeres of senescent cells^24^. Telomeres are nucleoprotein complexes pinned to the tail of linear chromosomes, consisting of tandemly repeated DNA sequences (5′-TTAGGG-3′) and shelterin, a protein complex that protects DNA tails from many enzymes involved in DNA processing and shortens with cells division^25^. Shelterin, a six-protein complex that includes TERF1, TERF2, POT1, T1N2, TPP1 and RAP1GAP, maintains telomere integrity^26^. TRF2–RAP1GAP works to prevent ATM activation to maintain chromosome integrity^27^. Telomerase (TERT), a reverse transcriptase that can repair shortened telomeres, is less common in somatic cells and more prevalent in cancer cells^28^. In telomerase-deficient mouse models, telomere shortening has been shown to have tumor-suppressive effects^29,30^. In colorectal carcinoma tissue, the expression of human telomerase and telomere length can distinguish cancerous tissue from adjacent tissue, with longer telomeres being associated with a worse prognosis^31^.

The senescence process is very complex and lacks a single specific and unique marker, leading to the proposal of CS as a collective phenotype of multiple effectors rather than a single entity^32^. In other words, senescence is a syndrome manifested by a combination of related cellular changes, including chromatin reorganization, gene expression profiles, secretome and metabolic pathways^33^. Here, we defined an index called “senescence signature” through analyzing CS gene expression profiles. This index serves as a reliable indicator of pan-cancer prognosis. In addition, this indicator was closely correlated with the immune characteristics of tumors, multi-omics alterations, telomere changes, and significantly predicted the immunotherapy response of various cancers, thereby guiding tumor treatment. We developed a new CS prediction model and established a portal website to apply this prediction model to predict the prognosis of patients with pan-cancer (https://bio-pub.shinyapps.io/cs_nomo/).

## Results

### The CS patterns effectively distinguish between tumor and tumor-adjacent normal tissues across pan-cancer types

We identified a co-expression module of genes, referred to as CS patterns, that are highly related (Pearson r > 0.5) to CS through WGCNA. This module includes 25 CS-related protein-coding genes, 2 lncRNAs and 32 miRNAs (Fig. S1A). Most of these CS-related protein-coding genes and extensive miRNAs interact within the co-expression regulation network (Fig. S1B). In the comparison of differential expression between adjacent normal tissues and tumor tissues across 16 different tumor types, many miRNAs were more highly expressed in tumor tissues, except in thyroid cancer (THCA) and pancreatic adenocarcinoma (PAAD) (Fig. S1C). At the same time, some CS inhibitory genes which play a key role in genome stability and cell cycle, including TACC3, BRCA1, E2H2, FOXM1, KIAA1524, AURKA and CDK1, showed similar expression patterns to miRNAs (Fig. S1C). This suggests that high expression of CS inhibitory genes would drive tumor progression, while miRNAs may exert a similar influence on tumor progression by down-regulating CS-induced genes. The area under the curve (AUC) of distinguishing between tumor and normal tissues using CS patterns exceeded 85% in most cancers (9 of 14) (Fig. S2A), suggesting that the CS patterns can discriminate between tumor and normal samples. We also gained the first two principal components (PCs) from pairs of tumor and normal tissues via the CS patterns and found that the two PCs were completely distinguishable in uterine corpus endometrial carcinoma (UCEC), colon adenocarcinoma (COAD), and kidney renal clear cell carcinoma (KIRC) (Figs. S2B-I, S3A-F), indicating that the CS patterns had a strong differential diagnostic role among the three cancers. We also analyzed the CS-related somatic genes mutation status in 33 cancers (Fig. S4). The top genes with the highest average mutated frequency were TP53, EPHA3, PDZD2, NOTCH3, and MYLK, which are involved in cell proliferation. It is missense mutation that was the most common form of mutation. These findings hinted the CS genes may be prone to missense mutations that affect cell proliferation during the process of senescence.

### Senescence signature could reflect intertumor and intratumor type heterogeneity and classify patients’ survival prominently in pan-cancer

**Fig. 1:**
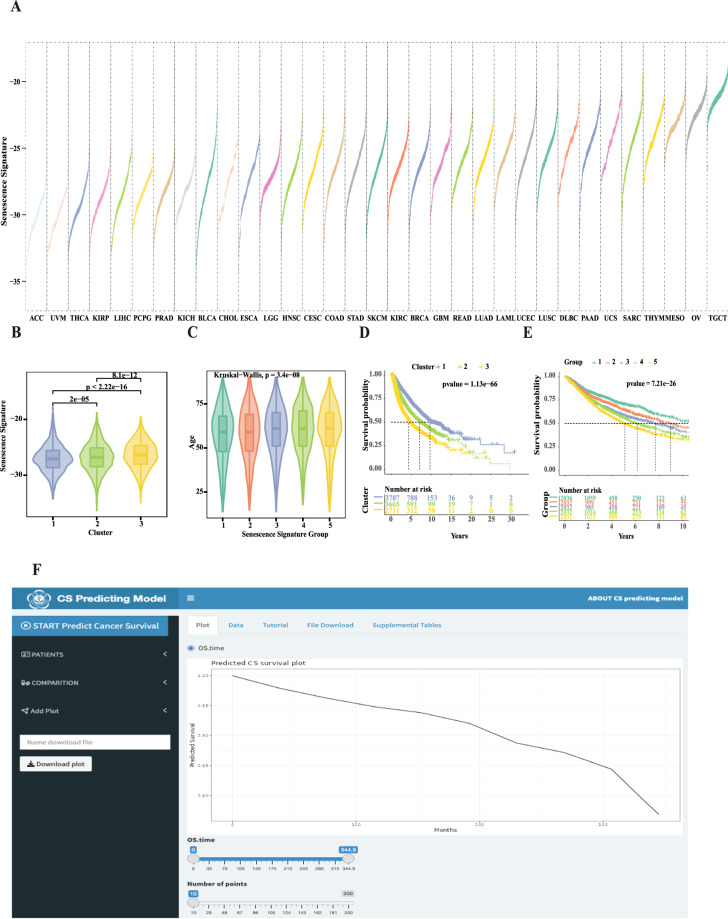
Landscape of senescence signature and the prediction of prognosis in TCGA pan-cancer. (**A**) Senescence signature across 33 TCGA tumor types, ordered by median. (**B**) The senescence signature increased among CS clusters 1–3 in pan-cancer (Wilcoxon test, all P < 0.0001). (**C**) Comparison of ages between CS groups defined by senescence signature score percentile in pan-cancer (Wilcoxon test, all P < 0.0001). (**D,E**) Kaplan-Meier curves of overall survival among 3 CS clusters (log-rank test, P = 1.13e-66) and 5 CS groups (log-rank test, P = 7.21e-26) in pan-cancer. (**F**) We developed a new CS prediction model and established a portal website to apply this prediction model to predict the prognosis of patients with pan-cancer (https://bio-pub.shinyapps.io/cs_nomo/).

To characterize the senescent microenvironment, we calculated the senescence signature scores for 33 types of tumors in the TCGA cohort to represent the degree of senescence (Fig. 1A). There were differences in the distribution of senescence signature scores within the same cancer cohort, as well as variations in the median senescence signature scores across different cancer cohorts. Germ cell tumors, such as testicular germ cell tumors (TGCT) and ovarian serous cystadenocarcinoma (OA), had the highest senescence signature scores, whereas adrenocortical carcinoma (ACC) had the lowest. These results suggest significant intertumor and intratumor heterogeneity in CS levels across pan-cancer types. Pan-cancer patients were divided into 3 CS clusters by K-means algorithm (Fig. S5A-B). The distinct CS clusters corresponded to different senescence signature scores (Fig. 1B, P < 0.0001), with CS cluster 1 having the lowest and CS cluster 3 the highest scores. Compared with CS cluster 1, CS clusters 2 and 3 had markedly worse OS (21 of 26), disease-specific survival (DSS) (16 of 22), and progression-free interval (PFI) (17 of 31) in most cancers (Fig. S6A-C, Cox regression analysis). The 3 CS clusters (Fig. 1D, P = 1.13e-66) and the 5 CS groups (Fig. 1E, P = 7.21e-26) were able to significantly classify overall pan-cancer patients’ survival, with higher senescence signature scores predicting worse prognosis. Compared with CS cluster 1, CS clusters 2 and 3 had a higher proportion of stage III and IV cancers (Fig. S7). The ages increased among CS clusters 1 to 3 (Fig. S7, P = 4.7e-08) and senescence signature score group 1 to 5 (Fig. 1C, P = 3.4e-08), indicating that the senescence signature effectively reflects aging. Additionally, we developed a new CS prediction model and established a portal website to apply this model to predict the prognosis of patients with pan-cancer (https://bio-pub.shinyapps.io/cs_nomo/) (Fig. 1F).

### Higher senescence signature was associated with higher genomic instability and proliferation in pan-cancer

**Fig. 2:**
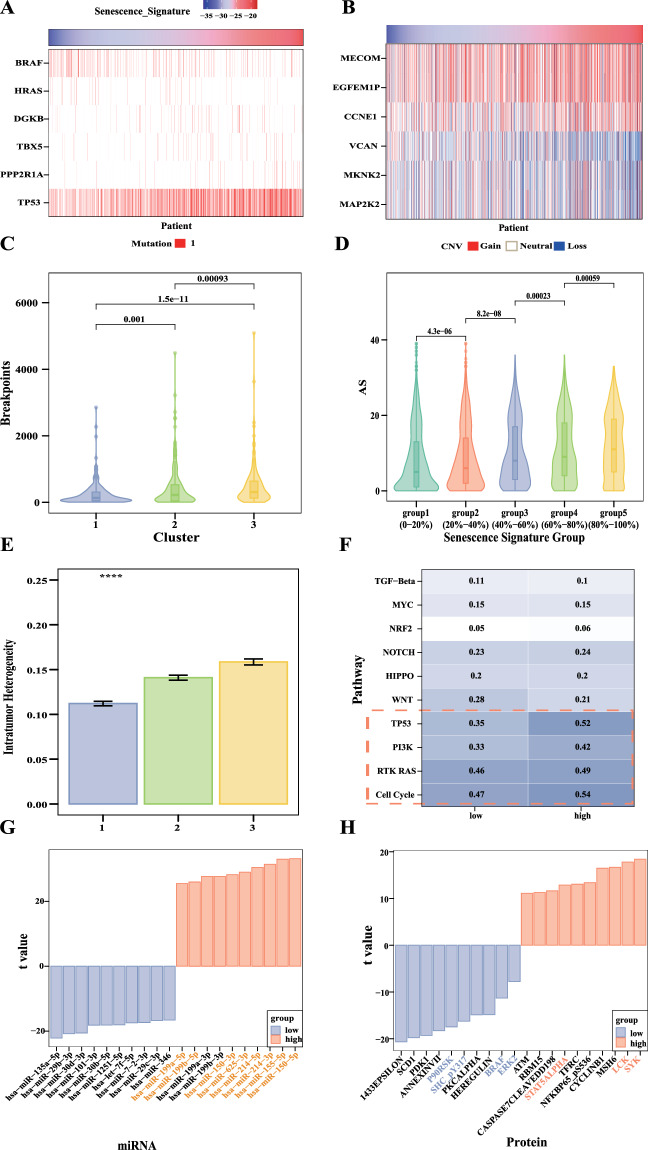
High senescence signature was associated with higher genomic instability in pan-cancer. (**A,B**) Heatmaps showing the notable associations of SNVs and CNVs with senescence signature in pan-cancer (Mann-Whitney U test, all P < 0.01). (**C**) Level of genomic breakpoints rose among CS clusters 1–3 of pan-cancer (Wilcoxon test, all P < 0.001). (**D**) Aneuploid score increased among the CS groups 1–5 of pan-cancer (Wilcoxon test, all P < 0.001). (**E**) Level of intratumor heterogeneity rose among CS clusters 1–3 of pan-cancer (Kruskal-Wallis test, P < = 0.0001). Following symbols were used to indicate statistical significance. (*P < = 0.05; **P < = 0.01; ***P < = 0.001; ***P < = 0.0001). (**F**) Differences in pathway mutation fraction between high and low senescence signature groups. (**G,H**) Bar plots showing miRNAs and proteins with statistically different expression between high and low senescence signature groups. The downregulated miRNAs/proteins of top 10 t value features were represented by blue, while upregulated miRNAs/ proteins of top 10 t value features were represented by pink. Yellow indicates miRNAs involved in the MAPK signaling pathway, blue indicates proteins involved in PTK-RAS pathway, and pink indicates proteins related to immune regulatory pathways.

We explored the associations between the senescence signature and genomic instability to establish its relationship with carcinogenesis. We found notable associations between SNVs and copy number variations CNVs with senescence signature in pan-cancer (Mann-Whitney U test). For SNVs, we found frequency of SNV occurrence increased in genes that inhibit cell proliferation, such as TP53 and PPP2R1A, and decreased in MAPK signaling pathway genes, such as BRAF and HRAS, with increasing senescence signature scores (Fig. 2A, all P < 0.01). For CNVs, frequent deletions of MAPK signaling pathway genes (e.g., MAP2K2/MEK2 and MKNK2) and frequent amplifications of genes promoting cell proliferation (e.g., EGFEM1P and MECOM) were associated with increased senescence signature scores (Fig. 2B, all P < 0.0001). Besides SNVs and CNVs, the quantitative indexes of genomic instability include genomic breakpoints, aneuploidy, and intratumor heterogeneity, which tend to increase with higher senescence signature scores. In the 3 CS clusters, genomic breakpoint levels rose with increasing senescence signature score (Fig. 2C, all P < 0.001). Similarly, in the 5 CS groups, aneuploidy scores increased with higher senescence signature scores (Fig. 2D, all P < 0.001). In the 3 CS clusters, level of intratumor heterogeneity also rose with increasing senescence signature scores (Fig. 2E, all P < = 0.0001). Higher senescence signature was related to higher proliferation, lower silent mutation rate, lower nonsilent mutation rate and lower SNV neoantigens (Fig. S8A-B, all P < = 0.001). For signaling pathways with frequent genetic alterations, the TP53 pathway, cell cycle pathway, PTK-RAS pathway, and PI3k pathway showed higher alteration frequencies in the high senescence signature group than in the low senescence signature group, while Wnt pathway showed the opposite result (Fig. 2F). We further explored the differential expression of miRNAs and proteins between high and low senescence signature groups in pan-cancer. 8 of top 10 upregulated miRNAs were related to MAPK pathway (Fig. 2G). 4 of top 10 downregulated proteins were related to PTK-RAS pathway (P90-RSK, SHC, BRAF and ERK), while 3 of the top upregulated proteins were related to immune regulatory pathways (STAT5, LCK, SYK) (Fig. 2H). These results suggest the aforementioned pathways may play an undeniable role in the CS process.

### Higher senescence signature score was related to increased lymphocyte infiltration and higher pro-tumor immune infiltrate in pan-cancer

**Fig. 3:**
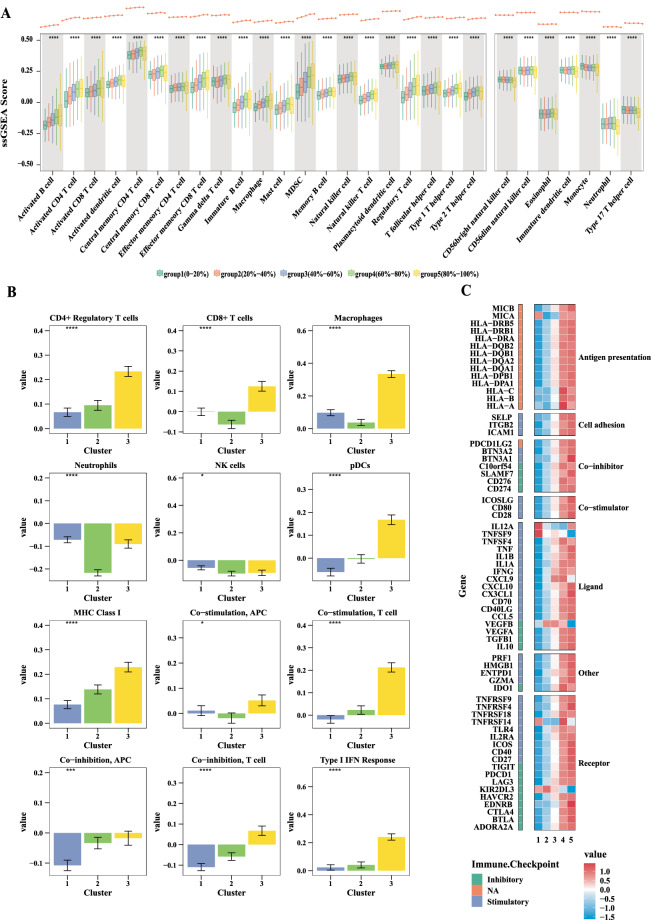
The immune landscape of CS in pan-cancer. (**A**) Boxplots showing the tumor microenvironment cell infiltration among 5 CS groups (Kruskal-Wallis test, all P < = 0.0001). Tumor infiltration scores for each tumor sample were calculated through ssGSEA. (**B**) Bar charts showing differences in tumor microenvironment cell infiltration among 3 CS groups (Kruskal-Wallis test, all P < = 0.0001). Cell-types were identified through CIBERSORT. (**C**) Expression of the immune regulatory pathways genes rose with increasing senescence signature score among 5 CS groups.

Further exploration of the influence of CS on the TME will play a role in understanding the mechanism of cancer promotion and immunotherapy. Higher senescence signature was related to higher leukocyte and stromal fraction in the 5 CS clusters (Fig. S9A, both P < 2.2e-16). A total of 28 immune categories showed significant differences among 5 CS groups (Fig. 3A, all P < = 0.0001) and 3 CS clusters (Fig. 3B, all P < = 0.05). Higher senescence signature score was related to increased lymphocyte infiltrate, except for type 17 T helper cells (Th17 cells), and higher pro-tumor immune infiltrate (such as Treg cell and MDSCs). The trends of eosinophil or neutrophil remained stable with increasing senescence signature score. In addition, the trend of type 1 IFN response was growing with increasing senescence signature score. Co-stimulation and co-inhibition of T cells, as well as co-inhibition of APCs, also showed a growing trend with higher senescence signature score. Expression of immune regulatory pathway genes rose with increasing senescence signature score in 5 CS groups (Fig. 3C). Specifically expressed genes of high senescence signature group were enriched in most immune regulatory pathways by KEGG enrichment analysis (Fig. S9B). These results suggest that one way CS promotes malignant tumor behavior is by regulating the immune cell components in the TME.

### Higher senescence signature was associated with longer telomere and higher TERT expression in pan-cancer

**Fig. 4:**
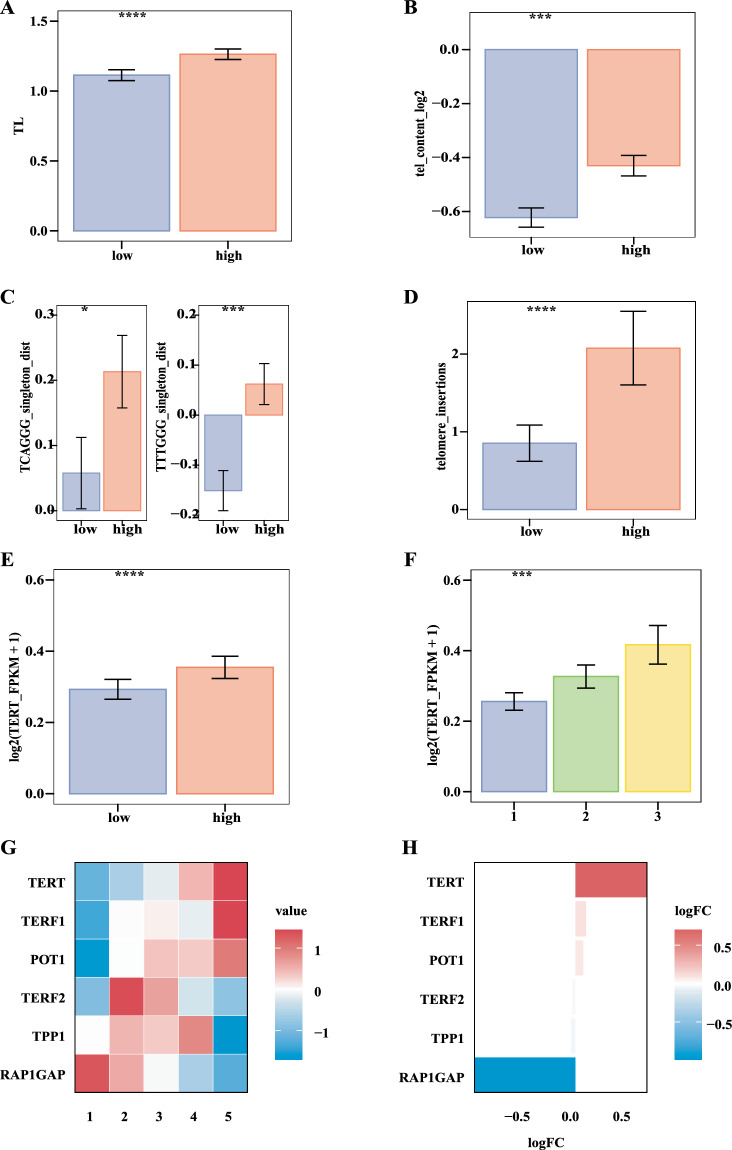
Higher senescence signature was associated with longer telomeres and higher TERT expression in pan-cancer. (**A,B,D,E**) Comparisons of telomere length, telomere content, telomere insertion and TERT gene expression, respectively, between the high senescence signature group (pink) and the low senescence signature group (blue) in pan-cancer (Wilcoxon test, all P < = 0.001). (**C**) Bar plots showing the differences of telomere variant repeats (TCAGGG and TTTGGG) between the high senescence signature group (pink) and the low senescence signature group (blue) (Wilcoxon test, all P < = 0.05). (**F**) TERT gene expression increased among CS clusters 1–3 (Kruskal-Wallis test, P < = 0.001). (**G**) Expression of shelterin genes among 5 CS groups. (**H**) Differences in expression of shelterin genes between the high senescence signature group and the low senescence signature group.

CS is closely related to changes in telomeres and telomerase. We found length of telomere was longer in group with higher senescence signature (Fig. 4A, P < = 0.0001), and telomere content (the ratio of telomere length between tumor and adjacent normal tissue in tumor-matched samples) was higher in these groups (Fig. 4B, P < = 0.001) in pan-cancer. The aberrant telomere variant repeats (TVRs), including TGAGGG and TTGGGG, were more prevalent in groups with higher senescence signature (Fig. 4C, test, both P < = 0.05). Targeted telomere insertion (TTI) was also higher in group with higher senescence signature (Fig. 4D, P < = 0.0001). TERT expression was elevated in groups (Fig. 4E, P < = 0.0001) and cluster (Fig. 4F, P < = 0.001) with higher senescence signature. With increasing senescence signature, TERT expression increased while RAP1GAP expression decreased (Fig. 4G-H). Our results suggested that CS affects tumor progression by altering telomere length and the degree of DNA damage on telomeres.

### Single-cell analysis revealed consistent evolutionary trends between malignant cells and immune cells at the CS level

**Fig. 5:**
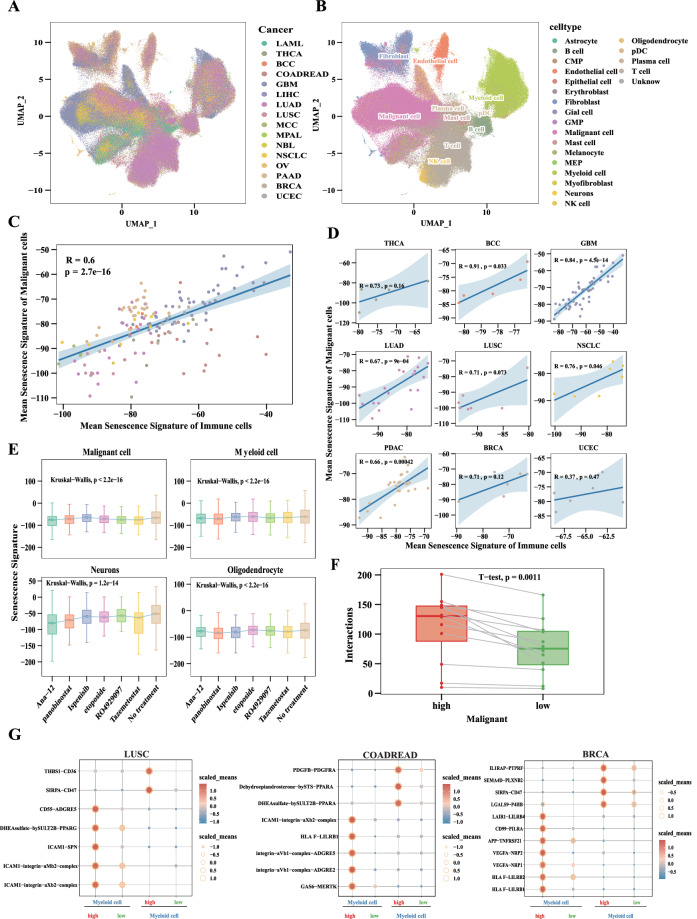
Single-cell analysis revealed malignant cells and immune cells have a consistent evolutionary trend at the CS level. (**A**) UMAP plot showing 539,350 cells from16 cancers. (**B**) UMAP plot categorizing 539,350 cells into 22 cell types. (**C**)The mean senescence signature of malignant cells was positively correlated with the mean senescence signature of immune cells, including T cells, B cells, plasma cells, NK cells, mast cells, pDC, and myeloid cells in pan-cancer. (**D**) In 9 single cancers, the mean senescence signatures of malignant cells were positively correlated with the mean senescence signatures of immune cells, including T cells, B cells, plasma cells, NK cells, mast cells, pDC, and myeloid cells. (**E**) After using 7 anti-cancer drugs to treat glioblastoma (GBM), senescence signature of malignant cell and components of TME (myeloid cells, neurons and oligodendrocyte) decreased (Wilcoxon test, P < 2.2e-16). (**F**) The higher degree of interaction between malignant cells and immune cells in the high senescence signature group (t test, P = 0.0011). Each of these dots represents a cancer species. (**G**) Interaction analysis showing enriched receptor-ligand pairs in myeloid cells and malignant cells between high and low senescence signature groups in cancers. The left panel shows the action of malignant cells on myeloid cells, while the right panel shows the action of myeloid cells on malignant cells.

Single-cell analysis UMAP plots showed 539,350 cells from 16 cancers (Fig. 5A) and categorized them into 22 cell types (Fig. 5B). The mean senescence signature of malignant cells was positively correlated with the mean senescence signature of immune cells, including T cells, B cells, plasma cells, NK cells, mast cells, pDC, and myeloid cells in pan-cancer (Fig. 5C). Similarly, in 9 specific cancers, the mean senescence signatures of malignant cells were positively correlated with the mean senescence signatures of above-mentioned immune cells (Fig. 5D). After treating glioblastoma (GBM) with seven different anti-cancer drugs, senescence signature of malignant cell and other cell populations in TME (including myeloid cells, neurons and oligodendrocyte) displayed a consistent downward trend (Fig. 5E, P < 2.2e-16). There was a higher degree of interaction between malignant cells and immune cells in the high senescence signature group at the pan-cancer level (Fig. 5F, P = 0.0011). Moreover, a higher degree of interaction was observed between malignant cells and myeloid cells in the high senescence signature group across many human cancers (Fig. 5G and Fig. S10A). The receptors on myeloid cells receive a rich range of proangiogenic factors from malignant, such as VEGFA and ICAM1.

### The essentiality of each CS genes had distinction, and alternated at different levels of senescence through CRISPR genome screening at the pan cancer level

We used a CRISPR genome screening dataset to verify the essentiality of CS genes and analyze the relationship between the essentiality of CS-related genes and senescence signature in pan-cancer. Interestingly, although both growth-inhibiting and growth-promoting genes were involved in the senescence process, it seemed that growth-promoting genes account for a greater proportion. Most of these genes were cell cycle-related genes, including CHEK1, CDK1, RUVBL2, PSMD14, SUPT5H, RBX1, and AURKA, across 28 cancer types (Fig. S11A). The essentiality of CS-related genes varied between high and low senescence signature groups in a tumor-type-specific manner (Fig. S11B). We found 12 CS-related genes whose essentiality had significant differences between high and low senescence signature groups of pan-cancer. In high senescence signature group, the CS-related genes with higher essentiality included CDKN2B and ANP32E, whereas the CS-related genes with lower essentiality included ZFP36L1, GAB1, and BRAT1. These findings may indicate that the essentiality of CS-related genes differentiates at different CS levels (Fig. S12A).

### Senescence signature had the potential to predict immunotherapy and targeted therapy response

**Fig. 6:**
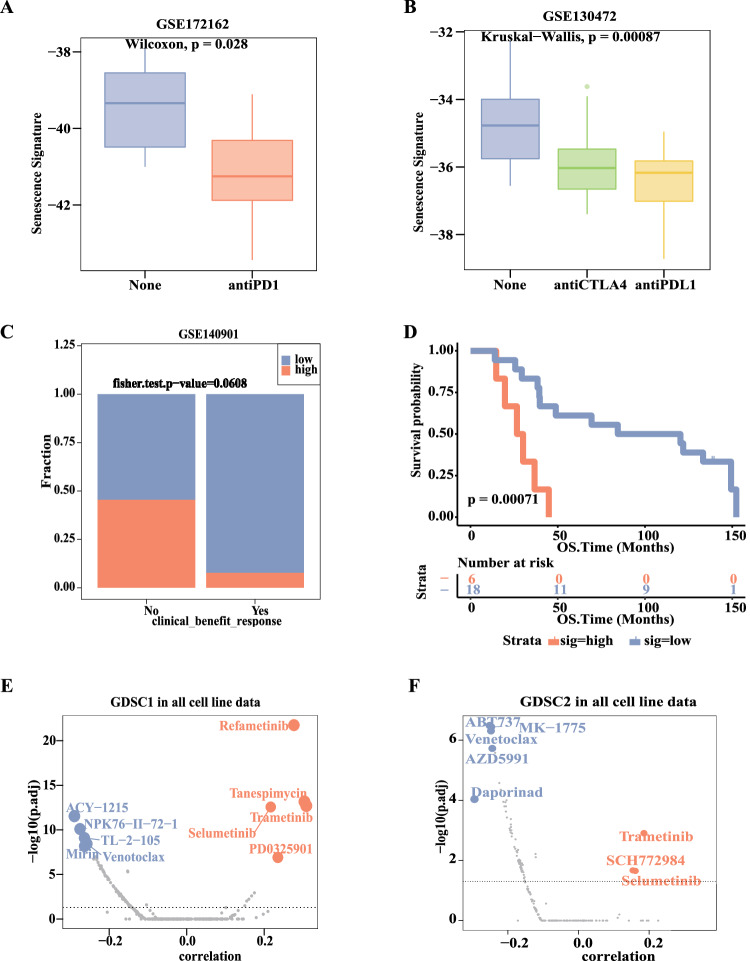
Senescence signature could predict immune therapy and targeted therapy response. (**A**) Comparison of senescence signature scores between PD-1 blockade treatment and no treatment group in MC38 cells (Wilcoxon test, P < 0.028). (**B**) Differences of senescence signature score among different immunotherapy in mammary tumor with BRCA mutation mice (Kruskal-Wallis test, P = 0.00087). (**C**) The HCC patients with objective response after anti-PD-1/anti-PD-L1-based therapy divided into two groups with or without clinical benefit response. The differences in the proportion of high senescence signature between the two groups were tested by Fisher’s exact test, P = 0.068. (**D**) After anti-PD-1/anti-PD-L1-based therapy, OS of HCC patients with high senescence signature was worse than those with low senescence signature (log-rank test, P = 0.00071). (**E,F**) The correlation of senescence signature with drug sensitivity was performed with GDSC drug data in pan-cancer. Blue dots represent the 5 top drugs with a negative correlation and p < 0.05, red dots represent the top 5 drugs with a positive correlation and a p < 0.05.

Senescence signature, as an index of TME features, has the potential to predict responses to immunotherapy. In MC38 cells of mice from GSE172162 dataset, senescence signature of group with receiving anti-PD-1 therapy was found to be lower than group without anti-PD-1 therapy (Fig. 6A, P < 0.028). In mammary tumor with BRCA mutation mice from GSE130472 dataset, both senescence signature of groups receiving anti-PD-1 and anti-CTLA4 therapies were lower than those of groups without immunotherapy. (Fig. 6B, P = 0.00087). In hepatocellular carcinoma (HCC) patients from GSE140901 dataset, the proportion of high senescence signature of the group with clinical benefit response after anti-PD-1/anti-PD-L1-based therapy was significantly lower than the group without clinical benefit (Fig. 6C, P = 0.068). HCC patients with higher senescence signature predicted worse prognosis with PD-1/PD-L1 blockade immunotherapy (Fig. 6D, P = 0.00071). Compared with INF-β stimulation group, TNF-α and INF-γ stimulation had lower senescence signature across 6 cancer types from RTM28723893 of TISMO database^34^ (Fig. S13A). Meanwhile, we also found correlation between senescence signature and sensitivity of antineoplastic drugs. The correlation of senescence signature with drug sensitivity was performed using GDSC IC50 drug data in pan-cancer (Fig. S14A-B, all P < 0.05). Lower senescence signature was associated with lower IC50 among multiple MEK1/2 inhibitors or ERK1/2 inhibitors, such as trametinib, selumetinib, refametinib, PD0325901, SCH772984. Higher senescence signature was associated with lower IC50 values among multiple BCL-2 family inhibitors, such as venetoclax, ABT-737, and AZD5991 (Fig. 6E-F, all P < 0.05). The IC50 of venetoclax showed negative correlation with senescence signature scores in GDSC1 and GDSC2. The IC50 of both trametinib and selumetinib showed positive correlation with senescence signature scores in GDSC1 and GDSC2. Higher senescence signature scores were related to lower expression of MAPK signaling pathway genes (MAPK3/ERK1, MAPK1/ERK2, and MAP2K2/MEK2) and higher expression of anti-apoptotic BCL-2 family genes (BCL2A1, MCL1, and BCL2) (Fig. S15A-B). Genes targeted by up-regulated miRNAs were enriched in MAPK signaling pathway by KEGG enrichment analysis (Fig. S16). Patients with high senescence signature had low protein expression (BRAF and ERK2) in the MAPK signaling pathway (Figs. S17-18).

## Discussion

According to evaluated levels of CS in 10,510 tumors spanning 33 tumor types, we found intertumor and intratumor heterogeneity of CS within each tumor type. Meanwhile, senescence signature effectively reflected the prognosis of pan-cancer patients, with higher senescence signature scores correlating with worse prognosis. CS is a dynamic process driven by a variety of stressors that cause persistent DNA damage and genomic instability, which in turn promotes and maintains CS^35–39^. Studies have shown that senescence escapers exhibit a stem-like signature with high tumor-initiating potential^40^. In our research, high senescence signature groups represent these “senescence escapers”. We found higher senescence signature scores were related to older age, higher genomic instability, greater intratumor heterogeneity, higher proliferation, longer telomeres, and higher telomerase expression. Genomic instability, which drives heterogeneity, is not only a predisposing factor of cancer but also a marker of advanced cancer^41^. TRF2–RAP1GAP is important for maintaining chromosomal integrity by preventing ATM activation^27^. We found higher senescence signature scores were related to low expression of RAP1GAP, and verified that telomeres play a role in genome instability during CS. Consistent with our finding that patients with higher senescence signature and longer telomeres have worse clinical outcomes, previous studies have shown that expression of human TERT and telomere length can distinguish cancerous tissue from adjacent tissue, with longer telomeres being associated with worse prognosis^31^.

We revealed the evolutionary trend of malignant cells and immune cells at the CS level. While most single-cell analysis on the senescent TME focus on the intercellular heterogeneity of CS levels^42–44^, the linear relationship between malignant cells and immune cells at the CS levels has not been studied. Our single-cell analysis found a positive correlation in the senescence signature between malignant cells and immune cells. In addition, our results showed a higher degree of interaction between these cell types in the high senescence signature group. This may indicate that malignant cells and immune cells interact to achieve a consistent evolutionary trend during the progression of CS. Previous studies have also shown that senescent malignant cells can induce chronic senescent inflammation, promoting cancer progression by modulation of cytoplasmic chromatin-cGAS-STING pathway^45^. Our research supports this viewpoint. We also found that in the high senescence signature group, the interaction between malignant cells and myeloid cells involves proangiogenic factors such as VEGFA and ICAM1. This is consistent with previous reports that proangiogenic factors drive immunosuppressive macrophages in HCC^46^.

We found higher senescence signature scores were related to increased lymphocyte infiltrate (except for type 17 T helper cells), higher stromal cells infiltrate, and higher pro-tumor immune infiltrate (Treg cells and MDSCs) across 33 cancers. Although immune reaction of CS is activated because of higher lymphocyte infiltrate, higher infiltrate of stromal cells, Treg cells and MDSCs promotes immune escape and thereby drives tumor progression. The previous studies have shown that the immune reaction of CS is activated by a positive feedback regulation of the SASP factors such IL1, IL6, IL8, IFN-I, TGF and TNF^33,45,47–53^. Despite the past view that high lymphocyte infiltrate predicts a good prognosis, immunocyte plasticity within TME refuted this view. The recent study found that IFN-γ dominant subtype with high lymphocyte infiltrate predicts poor prognosis^54^. Lymphocyte infiltrate represents the adaptive immunity in cancer and also possesses great plasticity within the TME^55^. In addition, high Treg cells and MDSCs infiltrate enhances immune-evading mechanisms^56,57^. Senescent stromal cells have been proven to promote immunosuppressive immune infiltrates in mouse experiments by secreting IL-6, such MDSCs and Treg cells^9^. Cancer-associated fibroblasts (CAF) also can regulate myeloid and T cell infiltration and produce factors that influence immune cell differentiation and plasticity, driving immunosuppressive phenotypes^58^. Several drugs targeting the pro-inflammatory effects of SASP have been found in previous studies. NF-κB-mediated signaling plays a key role in the pro-inflammatory effects of SASP, while metformin and the mTOR inhibitor rapamycin can inhibit these effects of SASP by blocking NF-κB-mediated signaling^59,60^. Rapamycin has been reported to restrict the growth-promoting effects of senescent fibroblasts on prostate cancer cells^61^. We found TP53 mutation growing with increasing senescence signature. This finding is consistent with the observation that TP53 mutation and TP53-deficient HCC cells bypass senescence and secrete factors that stimulate macrophages into a tumor-promoting M2 state, promoting pro-tumor immune infiltrate^62,63^. We found higher senescence signature scores predict worse immune therapy response. This is consistent with the association of high pro-tumor immune infiltrate, greater intratumor heterogeneity, and lower SNV neoantigens with reduced efficacy of immunotherapy in most cancer types^64–66^.

Of note, we found that the lower senescence signature scores were associated with higher sensitivity of multiple MEK1/2 inhibitors or ERK1/2 inhibitors, such as trametinib, selumetinib, and refametinib. Conversely, higher senescence signature scores were associated with higher sensitivity to BCL-2 family inhibitors, such as venetoclax and ABT-737. With increasing senescence signature score, mutation decreased in MAPK signaling pathway genes, such as BRAF and HRAS, while CNVs frequently resulted in the loss of MAPK signaling pathway genes such as MAP2K2/MEK2. The expression of MAPK signaling pathway proteins, such as BRAF and ERK2, was lower in patients with high senescence signature than those with low senescence signature. Higher senescence signature scores were related to lower expression of MAPK signaling pathway genes (MAPK3/ERK1, MAPK1/ERK2, and MAP2K2/MEK2). In addition, genes targeted by up-regulated miRNAs were enriched in MAPK signaling pathway by KEGG enrichment analysis. The miRNAs negatively regulate gene expression, suggesting that the MAPK signaling pathway is down-regulated with increasing senescence signature score. In Kras-mutant pancreatic ductal adenocarcinoma mice, dual inhibition of MEK and CDK4/6 can induce CS and improve the efficacy of anti-PD-1 therapy^67^. In lung adenocarcinomas mice, inducing CS by dual inhibition of MEK and CDK4/6, followed by treatment with uPAR-targeting CAR T cells, selectively killed senescent cells and limited tumor growth^68^. We also found that the expression of apoptotic genes in the BCL-2 family (BCL2A1, MCL1, and BCL2) was higher in patients with high senescence signature than those with lower senescence signature. There is substantial evidence that the selective killing of senescent cells with BCL-2 family inhibitors is effective in the treatment of tumors. In a mouse xenograft model, navitoclax, a BCL-2 family inhibitor, combined with doxorubicin or etoposide, effectively promoted tumor elimination^69^. *In vitro* and in mouse xenografts, navitoclax can efficiently kill ovarian and breast cancer cells with PARP inhibitor-related CS^70^. *In vitro* and *in vivo* lung cancer models, the combined application of cisplatin and navitoclax can induce tumor regression^71^. ABT-737 has also been reported to remove senescent cells in the liver and promote normal hepatocyte regeneration^72^. In a word, MAPK signaling pathway and apoptotic mechanisms may play a key role in the process of CS. The senescence signature may effectively predict sensitivity of MEK1/2 inhibitors, ERK1/2 inhibitors, and BCL-2 family inhibitors.

In summary, this study depicted a comprehensive molecular landscape associated with CS and TME in pan-cancer. It suggested malignant cells and immune cells share a consistent evolutionary trend at the CS level, highlighting the critical roles CS may play in cancer outcomes and tumor therapy. We developed a new CS prediction model and established a portal website to apply this model to predict the prognosis of pan-cancer patients.

## Materials and Methods

### Data collection and preprocessing

Pan-cancer mRNA, miRNA, mutation datasets and clinical information were downloaded from TCGA^73^. These datasets came from 33 cancer types. We filtered out samples without clinical information such as age, clinical stage, and OS time. Finally, we acquired 10,510 samples containing mRNA and miRNA expression data simultaneously. And we collected 96 proteins coding genes that are associated with CS (Table S1)^74^. This research complies with the Ethics Committee of Peking University Third Hospital and the Declaration of Helsinki.

TCGA has made good efforts to minimize the batch effect of expression data for mRNA and miRNA. To reduce discrepancies of sequencing centers and platform differences, the expression data were additionally adjusted and batch-corrected. Genes with residual batch effects (~10% of genes) or with mostly zero reads were removed from the adjusted samples and replaced with NAs. Genes were adjusted using a novel algorithm called EB++; a variant of the Empirical Bayes / ComBat algorithm with training and testing features added^73^. Different cancer datasets from TCGA have been processed using a common set of bioinformatics pipelines, so that the data can be directly compared and combined (https://gdc.cancer.gov/about-gdc).

### WGCNA co-expression

We filtered lncRNAs and miRNAs that pearson correlation greater than 0.5 with CS-related genes. Then we used these genes to acquire co-expression module by applying WGCNA (v1.69). We got one co-expression module contained 25 CS-related genes, 2 lncRNAs and 32 miRNAs (Table S2) and visualized their relationship by Cytoscape (v3.9.1).

### Discrimination of tumor and normal samples

To find out these genes expression whether different between tumor and normal samples, we filtered out cancers that contain less than 3 tumor-normal pair samples, and 14 cancer types remained. Then we computed fold change as follow:

$${{\rm{FC}}}_{{\rm{i}}}=\frac{{\rm{mean}}({\log }_{2}({{\rm{Tumor}}}_{{\rm{i}}}+1))}{{\rm{mean}}({\log }_{2}({{\rm{Normal}}}_{{\rm{i}}}+1))}$$

Where i is each gene of 59 genes we acquired above. Then we use Principal Component Analysis to assess these whether these 59 genes could discriminate tumor and normal samples. And in most cancers, tumor and normal samples can be separated in PC1 and PC2. Each sample in PCA analysis would get a PCA scores^75^:

$${\rm{score}}={\rm{PC}}1+{\rm{PC}}2$$

These scores were evaluated by ROC, and the AUC scores of 9 cancers were larger than 0.9, suggested that these genes can discriminate tumor and normal samples well.

### Mutation frequency estimation

We summarized the tumor mutation frequency of CS-related genes in different cancer types. The frequency was computed as follow:

$${{\rm{freq}}}_{{\rm{i}}}=\frac{{{\rm{n}}}_{{\rm{i}}}}{{\rm{N}}}$$

Where i is CS-related gene, n is the number of samples with mutation in i-th CS-related gene, N is the total samples in each cancer type (Table S3).

### Unsupervised approach to define CS-related subtype

We used K-means clustering to cluster tumor samples in each cancer type based on 59 genes that obtained from WGCNA. For cancer types contained at least 3 tumor-normal pairs samples, we keep genes that fold change less than 0.67 or greater than 1.5 in tumor sample versus normal samples as k-means input. If the number of remaining genes less than 5, we used all 59 genes instead. Also, when a cancer type without normal samples, we used 59 genes to cluster samples. To ensure the stability of clustering results, we set iterations to 1000. And the number of clusters was determined by elbow method^76^.

### Senescence signature definition

To select senescence genes that correlated with survival, we used a cox regression model to acquire the HR value of each CS-related genes by adjusting age, clinical stage in each cancer type. We defined genes with HR less than 1 and p value less than 0.05 in at least 5 cancer types as protective factor, genes with an HR value greater than 1 and p value less than 0.05 as risk factors. After filtering above, 68 genes remained (Table S4). Then we used these genes to define senescence signature for each cluster. The senescence signature was defined by two steps. First, we got PC1 score for each gene by PCA analysis. Second, PC1 score multiplied by corresponded gene expression, and summed them up.

$${\rm{senescence\; signature}}=\sum {({\rm{PC}}1}_{{\rm{i}}}\times {{\rm{G}}}_{{\rm{i}}})$$

Where i is one of 68 potential signal genes, G is the expression of that gene. According to senescence signature score percentile, we divided tumor samples into 5 groups: group1 (0–20%), group2 (20%-40%), group3 (40%–60%), group4 (60%–80%), group5 (80%–100%).

### Survival analysis

We used R packages survival (v3.2-13) and survminer (v 0.4.9) to draw Kaplan-Meier survival curves among 3 CS clusters and 5 CS groups. We set lower overall survival (OS) time as cluster 3, and higher OS time as cluster 1. We applied cox regression model to verify whether higher cluster number with higher risk.

### Association of multi-omics datasets with senescence signature

To find out genome instability whether correlated with senescence score, we divided samples into 2 groups according to signature score levels. And used Mann–Whitney U test to obtain notably different single nucleotide variants (SNVs) and copy number variations (CNVs)^77^ (Table S5-6).

Besides SNVs and CNVs, the quantitative indexes of genomic instability include genome breakpoints, aneuploidy and intratumor heterogeneity. The double strand break (DSB) is an important manifestation of genomic instability, which was calculated by breakpoint rate^78^. Aneuploidy is an unbalanced number of chromosomes, which was defined as somatic copy-number alteration (SCNAs) of whole chromosomes and of chromosome arms. Aneuploidy was measured by aneuploid score^79^. Intratumor heterogeneity refers to the difference between classified offspring and parents as cancer cells continue to grow. Intratumor heterogeneity was measured by Intratumor heterogeneity score^80^. We also compared genome breakpoints, aneuploid score and intratumor heterogeneity score among CS clusters and CS groups and used Kruskal-Wallis to test significance.

We divided samples in two groups coded as “high” and “low” according to senescence signature. Pathway alteration fraction was computed as the fraction samples that had corresponded pathway alteration between these two groups^81^. And differentially expressed miRNAs (Table S7) and proteins (Table S8) were computed by limma (v3.46.0). We selected top 10 t value features to display.

### Association of TME with senescence signature

We obtained 28 immune categories from Charoentong *et al*.^82^, and used ssGSEA (v1.38.2) to calculate tumor infiltration scores for each tumor samples. Compared tumor infiltration score among CS groups by Kruskal-Wallis test. And we also compare the mean se of different types of immune cells among different CS clusters using Kruskal-Wallis test^54^.

### Association of telomere with senescence signature

Telomere is a crucial metric for CS. Related datasets were collected from Sieverling *et al*.^83^ and Barthel *et al*.^84^. We divided samples in two groups coded as “high” and “low” according to senescence signature. Then we compared different telomere-related information including telomere length, telomere content, singleton telomere variant repeats, telomere insertion and TERT gene expression between these two groups. And p values were computed by Wilcoxon.

### CRISPR genome screening of CS-related genes in pan-cancer

DepMap (Cancer Dependency Map) is a database used to screen potential therapeutic targets for cancer by utilizing RNAi and CRISPR-Cas9 technology. The survival dependency score of various cancer cell lines is referred to as CRISPRGeneEffect in DepMap, which represents the cell proliferation ability and activity of cell lines after knocking out specific genes through CRISPR-cas9. By constructing this indicator using the CERES algorithm^85^, a negative CRISPRGeneEffect of a gene means that knocking out of the gene inhibits the survival of a cell line, where a positive CRISPRGeneEffect of a gene means that knocking out of the gene promotes the survival and proliferation of a cell line. Cut off of 0.5 and - 0.5 are to define growth suppression genes and growth promotion genes. Therefore, it can be concluded that the essentiality score is inversely proportional to CRISPRGeneEffect. The color of the square represents CRISPRGeneEffect (Fig. S11A).

Through DepMap database, we can obtain 378 cell lines and 17387 genes without requiring the specific accession numbers from this public database (https://depmap.org/portal/), then calculated the mean of effect values of each CS related gene in each cancer type and evaluated the sums of effect value in genes levels and cancer types level separately. Then we used spearman to explore whether the senescence signature score correlate with effect value. And we also split cell lines into two groups according to their senescence signature score and find those genes that have different effect between high score group and low score group.

### Single-cell data analysis

Single cell transcriptome data and clinical data were downloaded from cancerSCEM^86^ (https://ngdc.cncb.ac.cn/cancerscem). Filter out cancer species that only contain one sample. Each sample was treated according to the cancerSCEM method, retaining cells with expressed genes between 201 and 5000, while filtering out cells with mitochondrial gene expression ratios greater than 10%. The data of individual cancer species have been batch corrected using Harmony (1.0)^87^ and dimensionality reduction (dims = 1:30) and clustering were performed using Seurat (4.1.0)^88^. Cell types were manually annotated based on cell specific marker (https://ngdc.cncb.ac.cn/cancerscem/documents)^89^. We used scanpy (1.9.2)^90^ analysis and Harmony for integration between multiple cancer species. In the end, we obtained 539350 cells from 16 cancer species and divided them into 22 cell types (Table S9).

At the same time, the senescence signature of each cell was calculated. Then, the Pearson correlation between the average senescence signature of malignant cells and the average senescence signature of immune cells in different samples was analyzed.

In order to investigate the differential degree of interaction of malignant cells and immune cells between the high and low senescence signature cells, we divided malignant cells in each cancer species into high and low groups based on senescence signature and conducted cell interaction analysis in each cancer species using CellPhoneDB (v4.0.0)^91^. Then, t-test was used to examine differential degree of interaction of malignant cells and immune cells between the high and low senescence signature groups.

### Association of immune therapy with senescence signature

To explore the association between senescence signature and immunetherapy response. We downloaded GSE172162 (https://www.ncbi.nlm.nih.gov/geo/query/acc.cgi?acc=GSE172162), GSE130472^16^ and GSE140901^92^ from TISMO database^34^. First, we computed senescence signature score in each samples and then compared the senescence signature score between samples without and with treatment. In GSE140901, we use surv_cutpoint function to select a cutoff of senescence signature score to divided samples into two group codes “high” and “low”. Then calculated the support ratio in samples with and without benefit response patients.

### Association of drug IC50 with senescence signature Landscape of CS pattern in TCGA pan-cancer

The IC50 of drug can predict pesticide effect. And different level of senescence signature may affect pesticide effect. We acquired different drug IC50 in different cell lines from GDSC (https://www.cancerrxgene.org/)^93^. After filtering cell lines that do not belong to any TCGA cancer type, we got GDSC dataset contained 738 cell lines and 345 drugs. And calculated the senescence signature of each cell line using gene expression profiles according to the above method. Then calculated spearman correlation between IC50 and senescence signature score (Tables S10–11). Adjusted p value was computed by Benjamini & Hochberg method. The full names of all cancers corresponding to the cancer abbreviations in the full text are displayed (Table S12). The source of the dataset for this article is detailed in Table S13.

### Supplementary information

Supplementary Figure

Supplementary Table

## Data Availability

Clinical information, mRNA, miRNA, mutation data sets and of 10510 samples across 33 cancer types were available in TCGA (https://portal.gdc.cancer.gov/). CS-related proteins coding genes were available in CellAge database (http://genomics.senescence.info/cells). We used the CRISPR genome screening dataset from DepMap database (https://depmap.org/portal/). We acquired different drug IC50 in different cell lines from GDSC (https://www.cancerrxgene.org/).

## Code availability

This article does not make use of any custom code.
