# Supplementary material for: Multi-omics and single cell characterization of cancer immunosenescence landscape
Source: Sci Data. 2024 Jul 7;11:739. doi: 10.1038/s41597-024-03562-z (PMC11228048; doi:10.1038/s41597-024-03562-z)
Supplement: Supplementary file 1 — Supplementary Figure [file 41597_2024_3562_MOESM1_ESM.pdf]

# Supplementary Figure 1

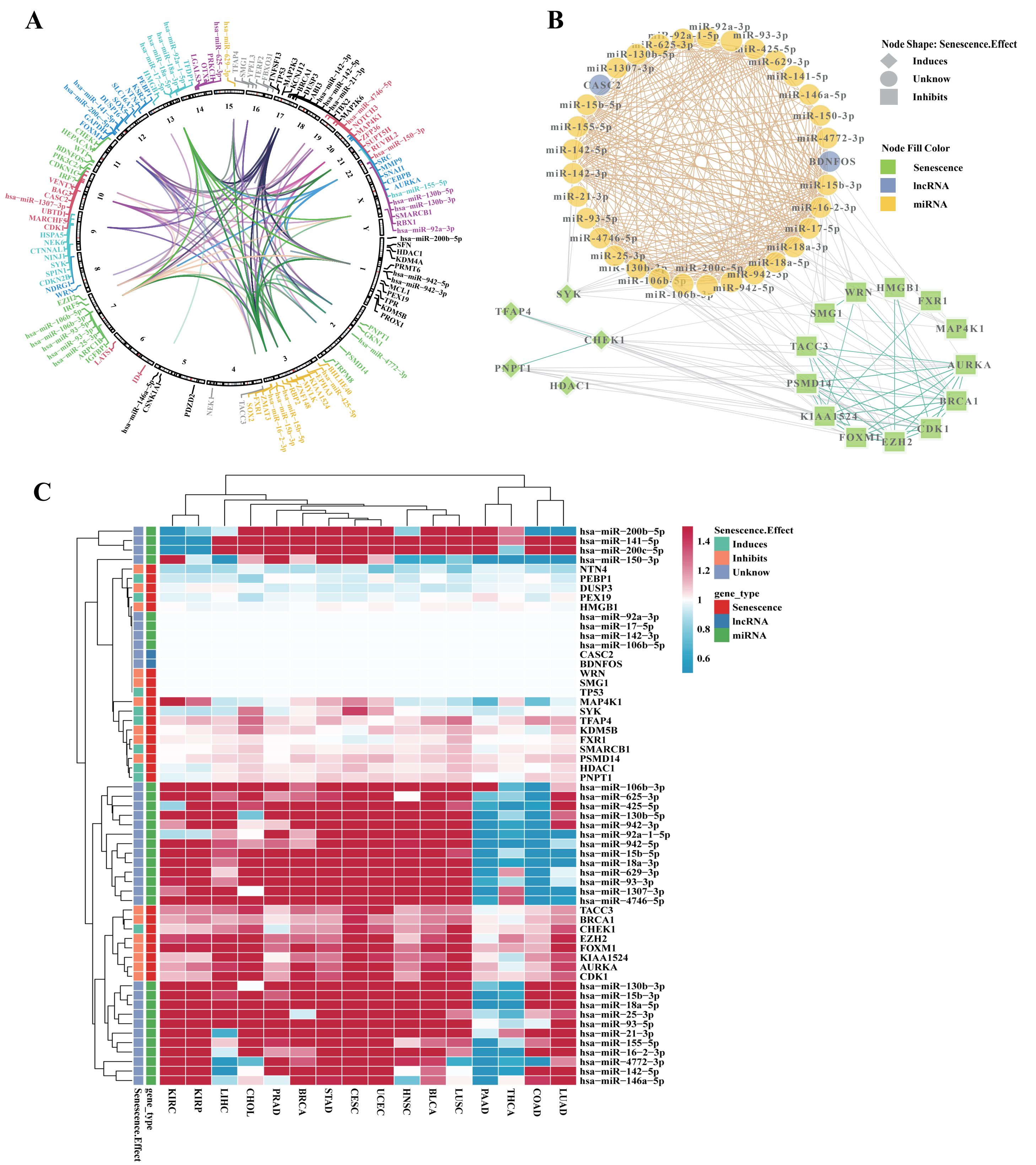


**Supplementary Figure 1. CS is closely related to pan-cancer.**

**(A) Circos plot and gene co-expression of the CS-related genes on the chromosome.**

**(B) Gene co-expression network of the CS-related genes. The gene pairs with Pearson r > 0.5 are considered to have co-expression correlation.**

**(C) Heatmap showing differential expression of CS-related genes between tumor and tumor-adjacent normal tissues across 16 cancer types.**

**(D) Discrimination analyses of the tumor and adjacent normal tissues in pan-cancer (cancer types with < 3 tumor-normal pairs filtered out) based on the CS-related genes expression.**

# Supplementary Figure 2

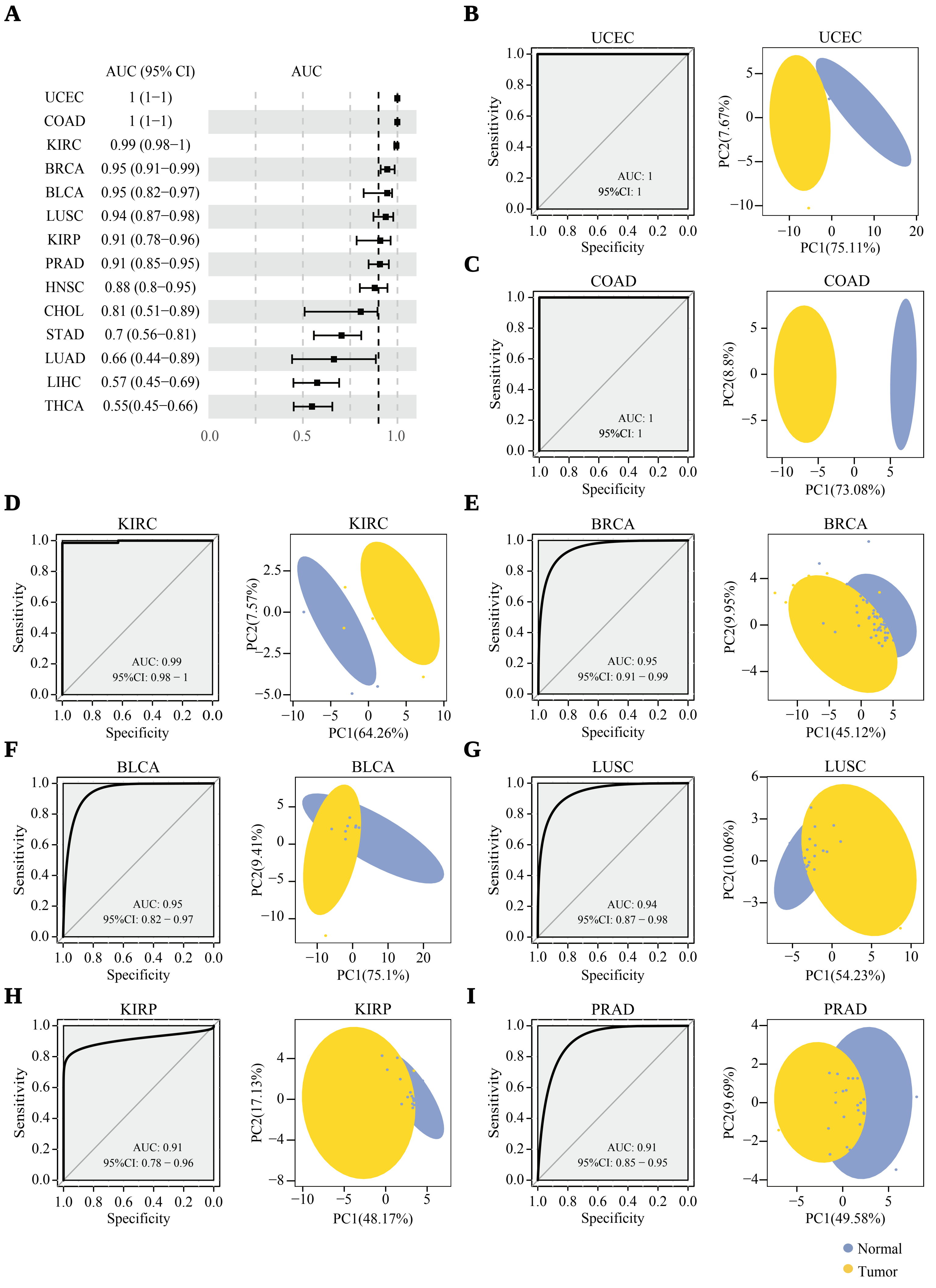

# Supplementary Figure 3

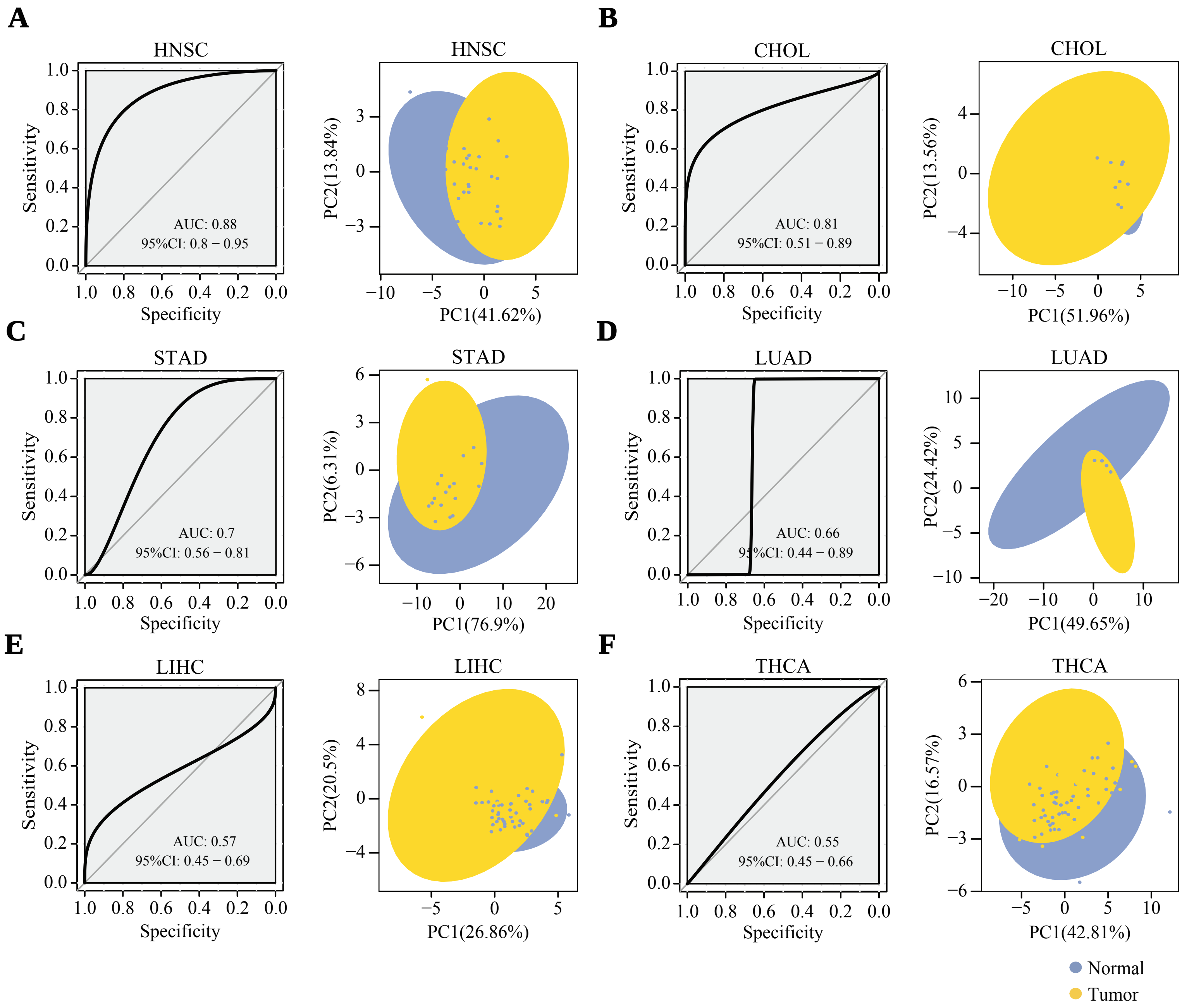


**Supplementary Figure 2. The CS patterns had good effect in distinguish between tumor and tumor-adjacent normal tissues across pan-cancer types.**
**(A) Discrimination analyses of the tumor and adjacent normal tissues in pan-cancer (cancer types with < 3 tumor-normal pairs filtered out) based on the CS genes expression. (B-I) Receiver-operating characteristic curves of predicted performance to discriminate from tumor and normal tissues and PCA plots of CS-related genes in tumors and adjacent normal tissues across 8 cancer types.**

**Supplementary Figure 3. Receiver-operating characteristic curves of predicted performance to discriminate between tumor and normal tissues and PCA plots of CS-related genes in tumors and adjacent normal tissues across 6 cancer types.**

# Supplementary Figure 4

**A**

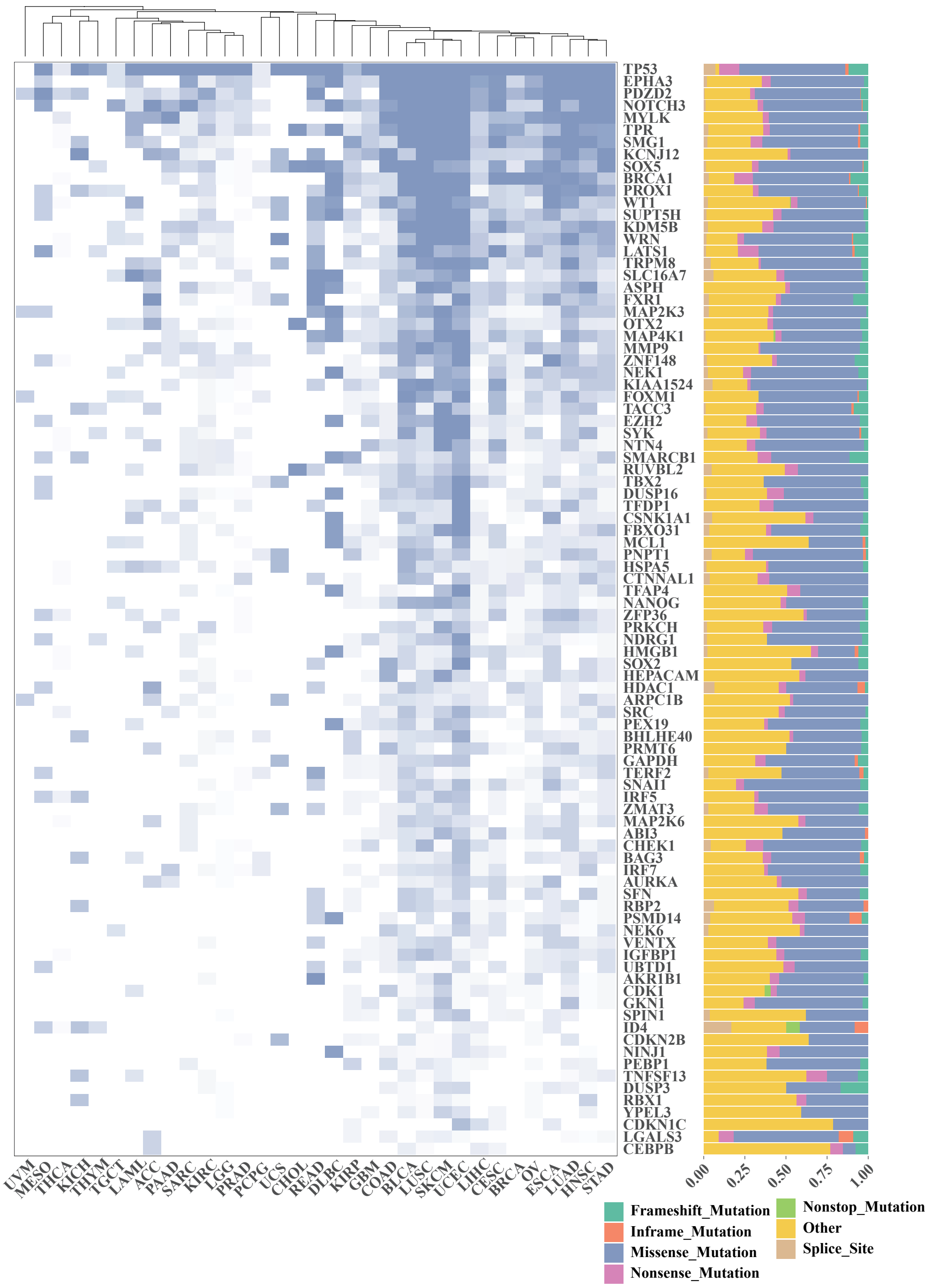


**Supplementary Figure 4. Heatmap of the somatic mutation frequency of the CS-related genes across 33 cancer types. The higher the mutation ratio, the bluer the color.**

# Supplementary Figure 5

A

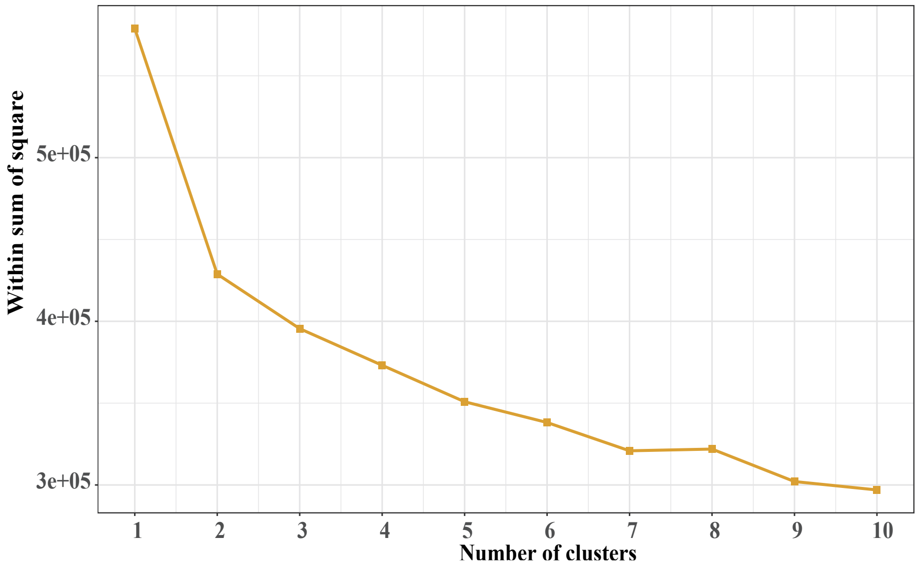


B

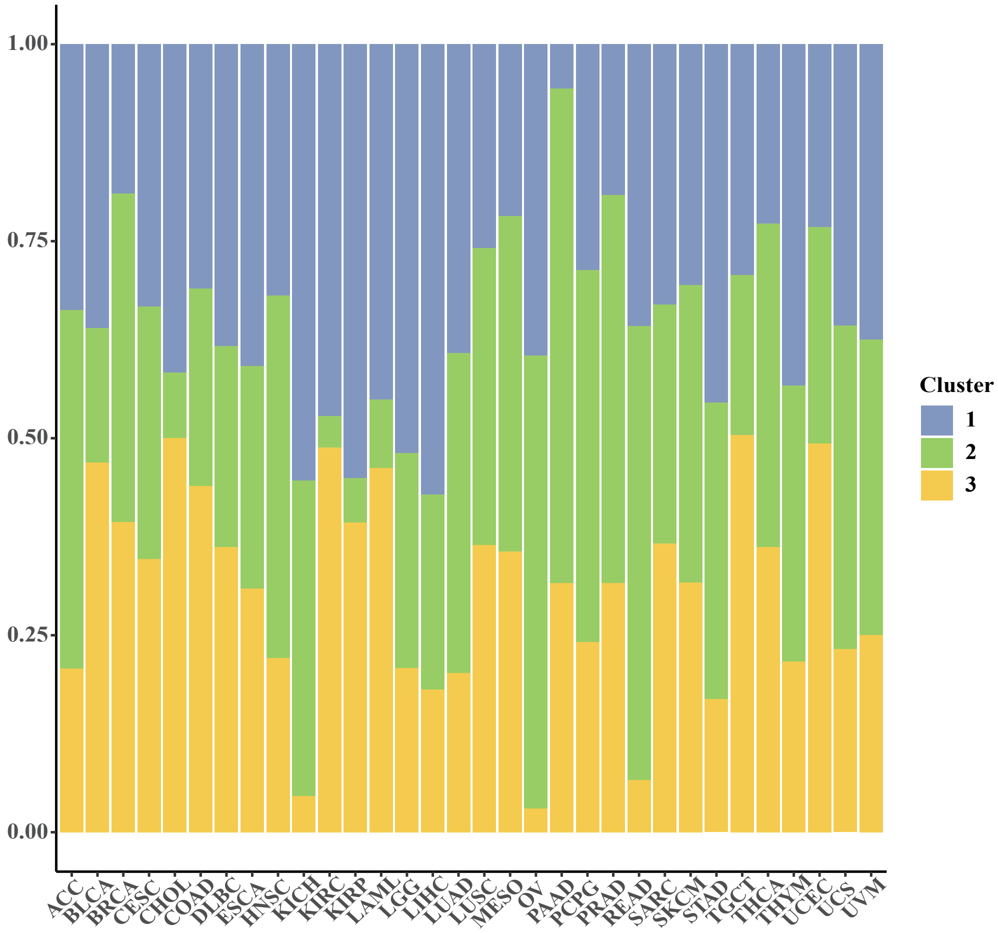


**Supplementary Figure 5. Pan-cancer patients were divided into 3 CS clusters by K-means algorithm.**

**(A) Determination of the number of clusters as 3 by the elbow method.**

**(B) Distribution of the CS clusters across each cancer type.**

# Supplementary Figure 6

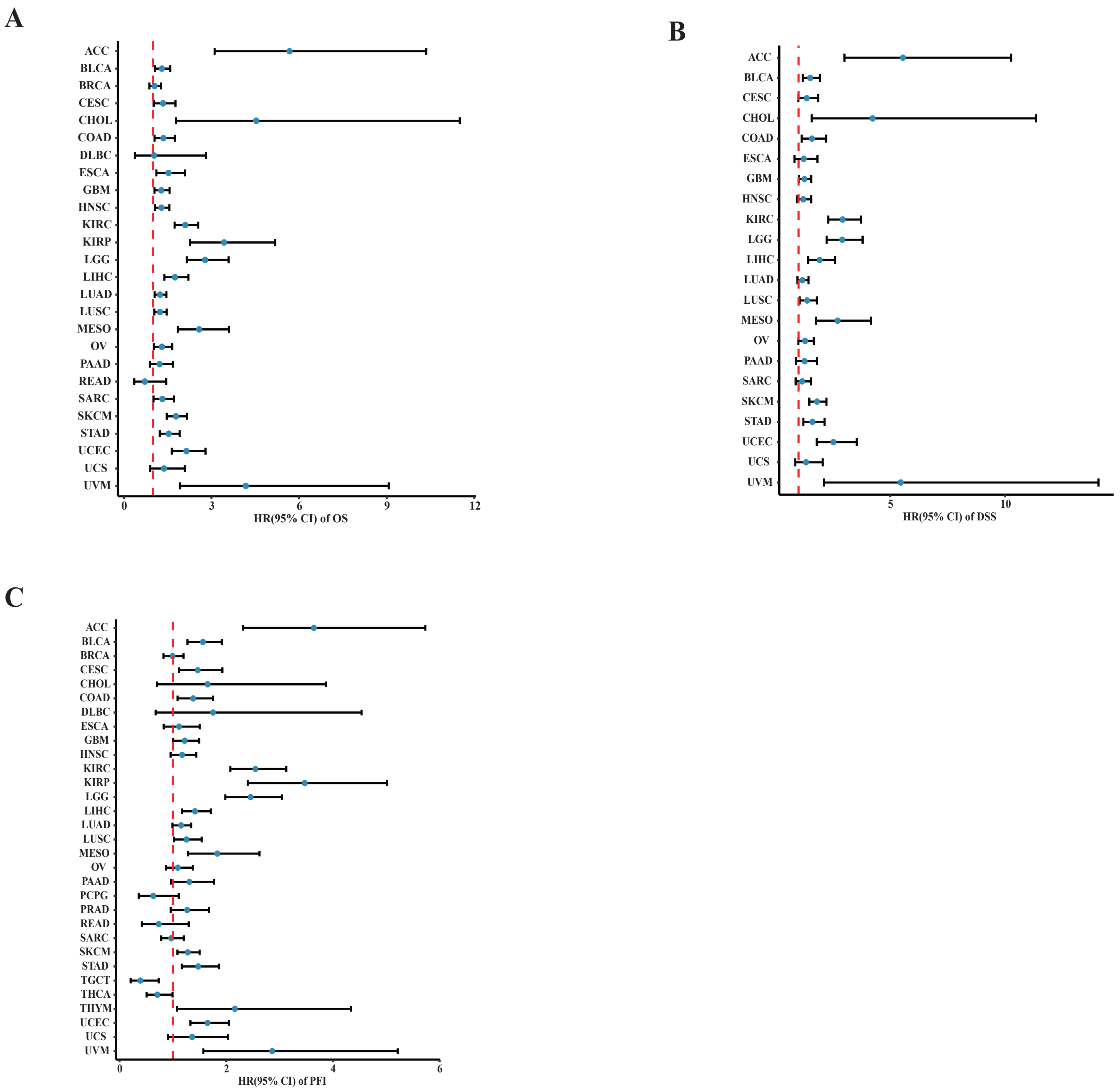


**Supplementary Figure 6. The CS clusters could predict prognosis of pan-cancer.**

**(A-C) Forest plots showing associations of the CS clusters with pan-cancer overall (OS), progression-free interval (PFI) and disease-specific survival (DSS). The hazard ratios (HR) are evaluated by the trend association (clusters 1-3) in the Cox proportional hazard models.**

# Supplementary Figure 7

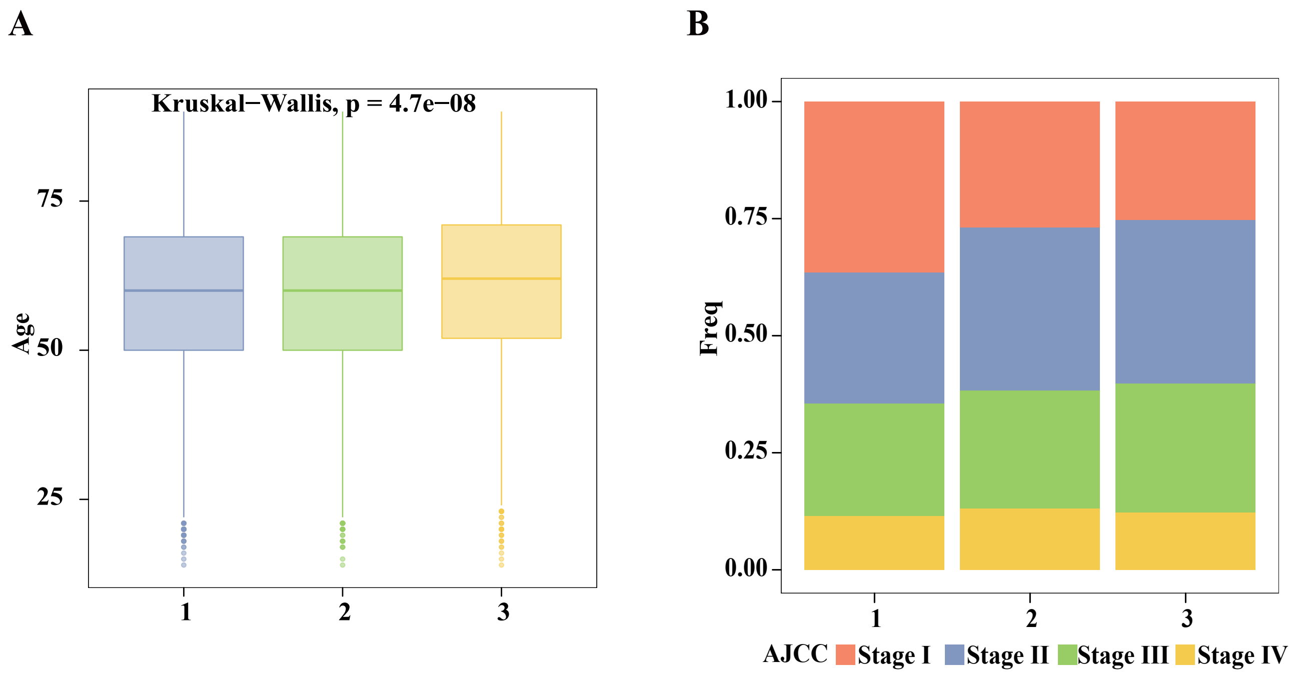


**Supplementary Figure 7. Difference in age and stage (I-IV) among 3 CS clusters of pan-cancer .**

**(A) The ages among 3 CS clusters of pan-cancer (Kruskal-Wallis test, P = 4.7e-08).**

**(B) The proportion of stage (I-IV) among 3 CS clusters of pan-cancer.**

# Supplementary Figure 8

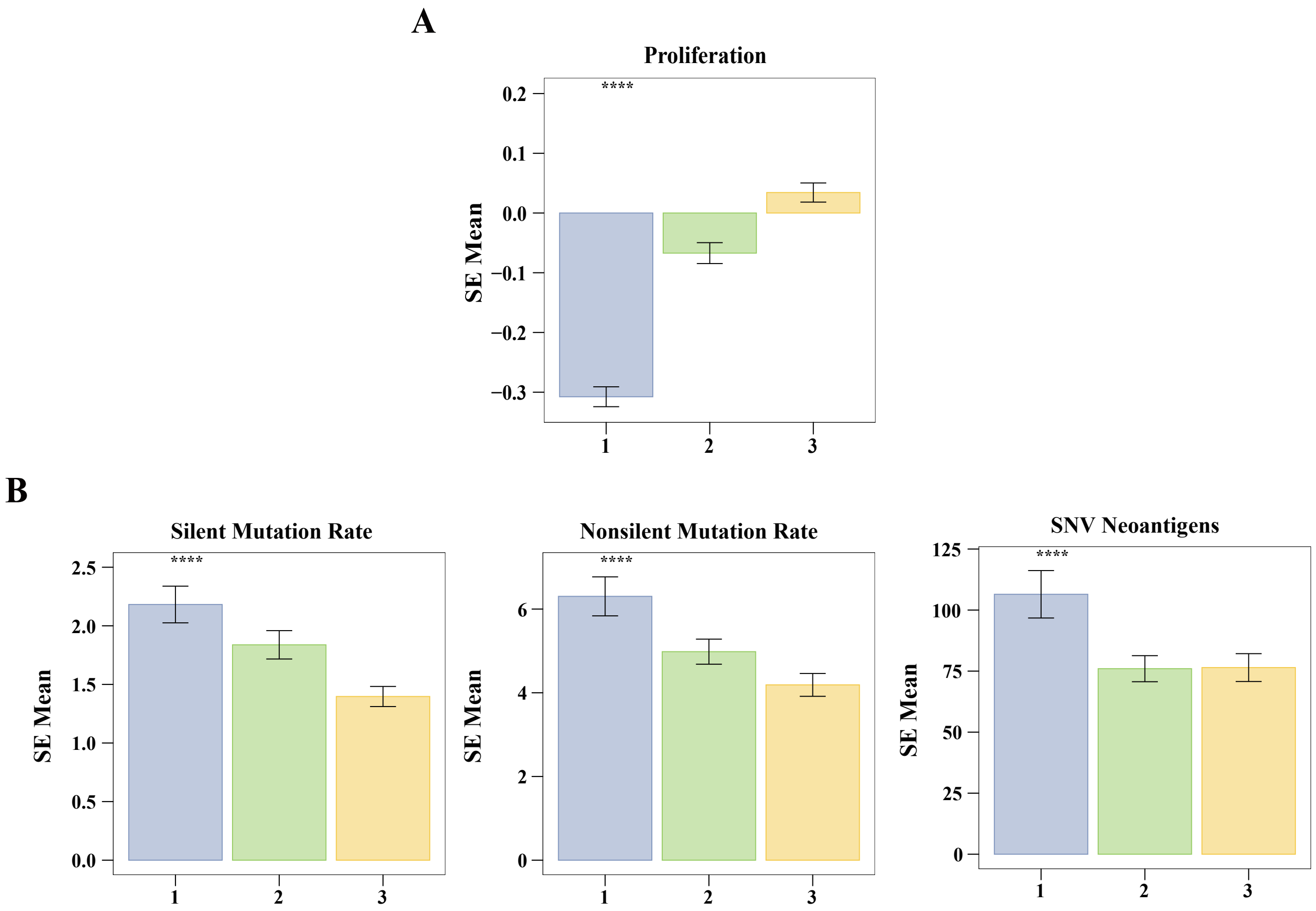


**Supplementary Figure 8. High senescence signature was associated with higher proliferation and lower SNV neoantigens in pan-cancer.**

**(A) Proliferation level rose among CS clusters 1-3 of pan-cancer (Kruskal-Wallis test, P <= 0.0001).**

**(B) High senescence signature was associated with lower silent mutation rate, nonsilent mutation rate and SNV neoantigens (Kruskal-Wallis test, all P <= 0.0001).**

# Supplementary Figure 9

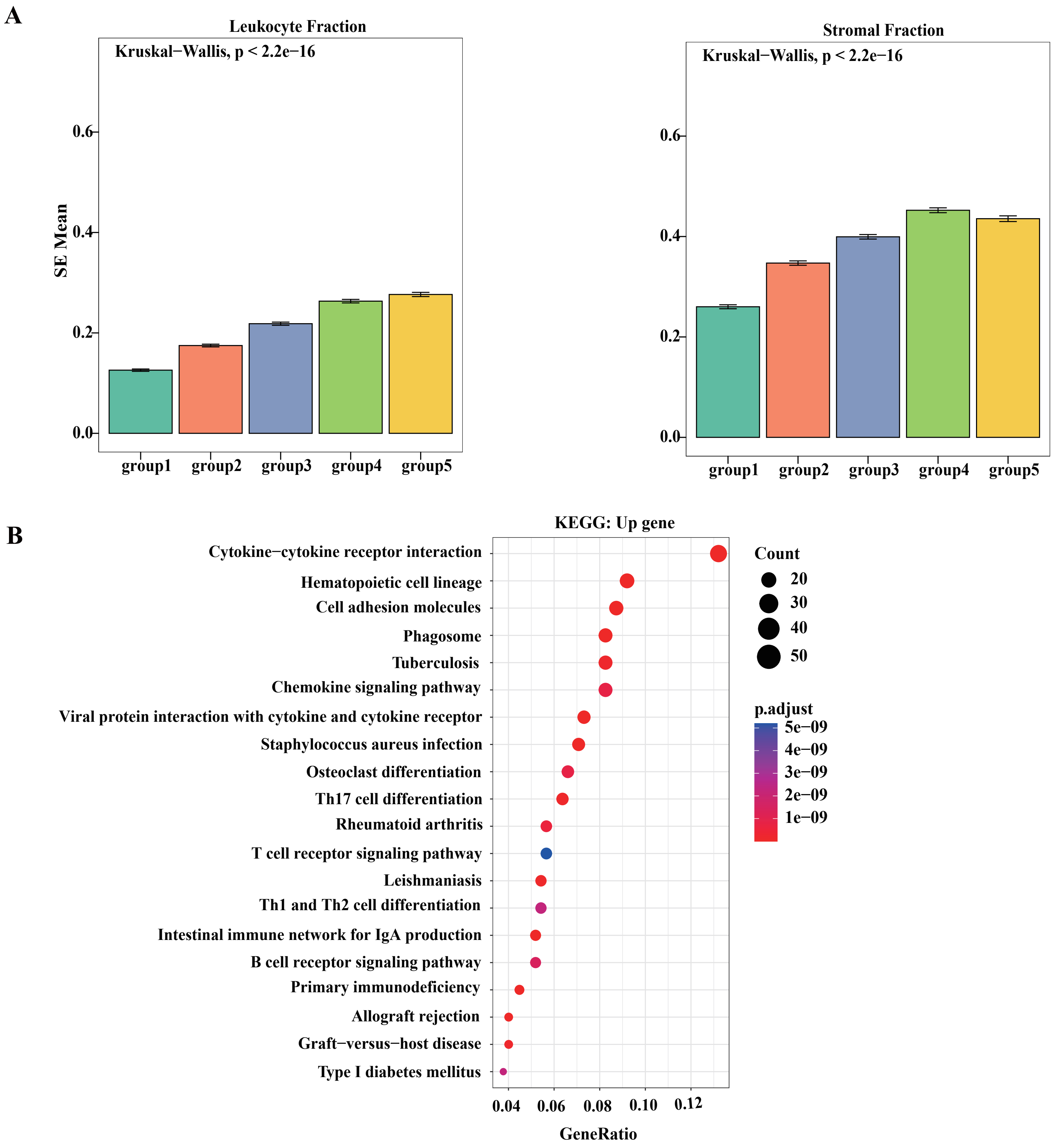


**Supplementary Figure 9. Association of TME with senescence signature in pan-cancer.**

**(A) Leukocyte fraction and stromal fraction among 5 CS groups of pan-cancer (Kruskal-Wallis test, both P < 2.2e-16).**

**(B) Upregulated expression genes of high senescence signature group were enriched in most immune regulatory pathways by KEGG enrichment analysis.**

# Supplementary Figure 10

A

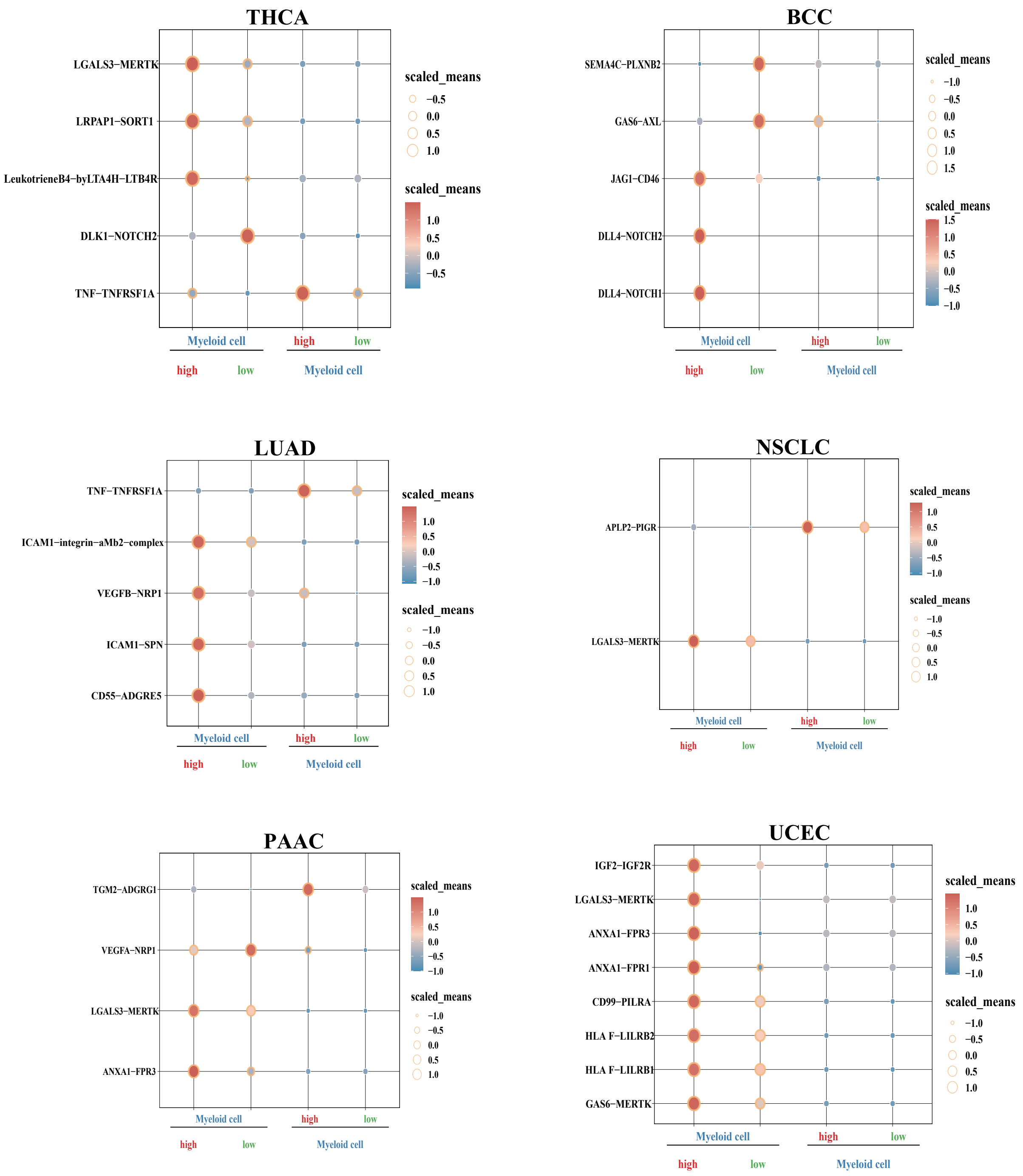


**Supplementary Figure 10. Interaction analysis showing enriched receptor-ligand pairs in myeloid cells and malignant cells between high and low senescence signature groups in multiple cancers. The left panel shows the action of malignant cells on myeloid cells, while the right panel shows the action of myeloid cells on malignant cells.**

# Supplementary Figure 11

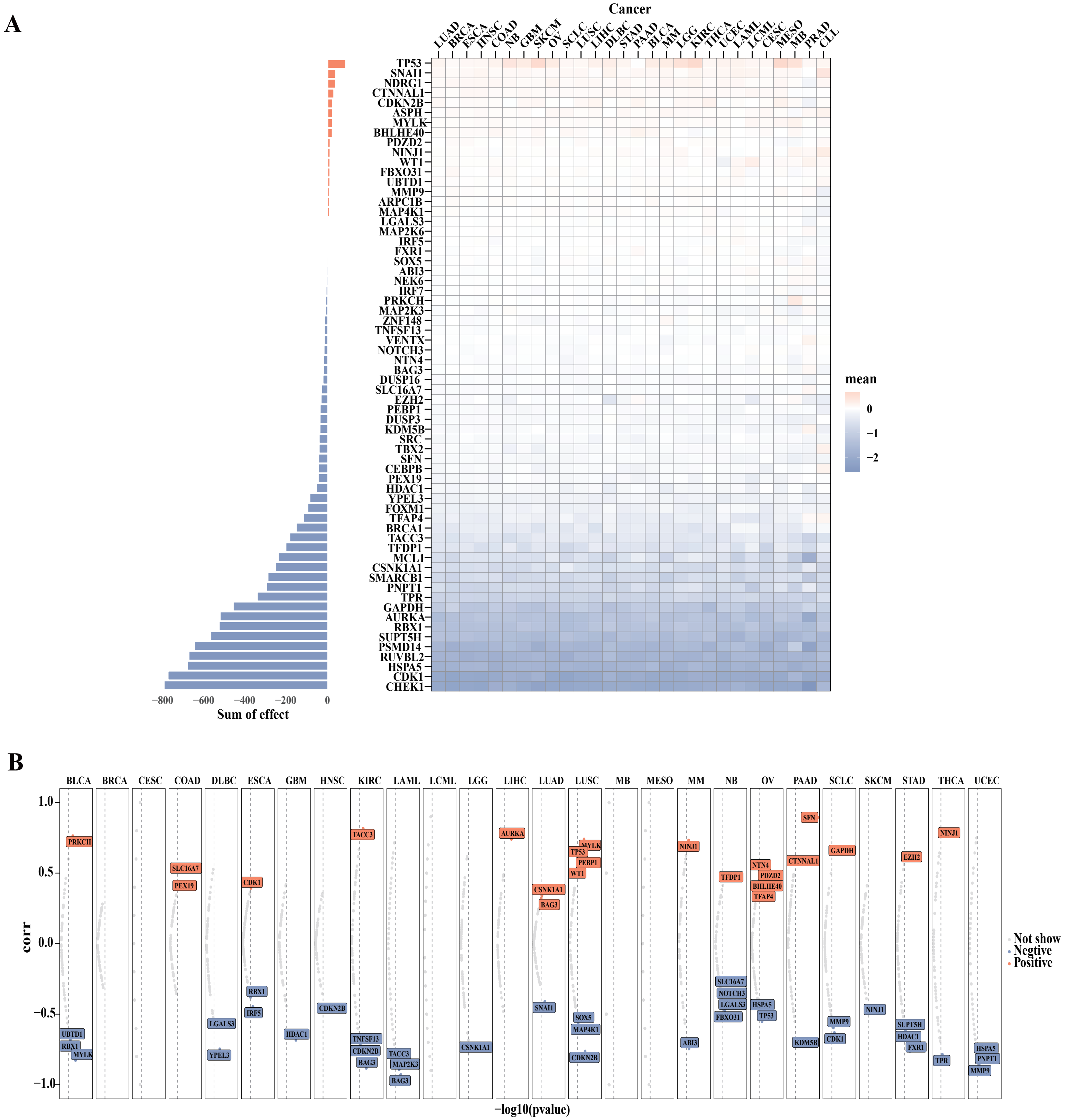


**Supplementary Figure 11. CRISPR genome screening of CS-related genes in pan-cancer.**
**(A) Comparison of gene effect of cell lines of 28 cancer types from DepMap Portal database. The gene effect is represented by the DEMETER2 dependency score based on data for a cell depletion assay. A lower DEMETER2 score indicates higher essentiality for a particular cell line.**
**(B) The correlation in essentiality of CS-related genes between high and low senescence signature groups across 28 cancer types.**

# Supplementary Figure 12

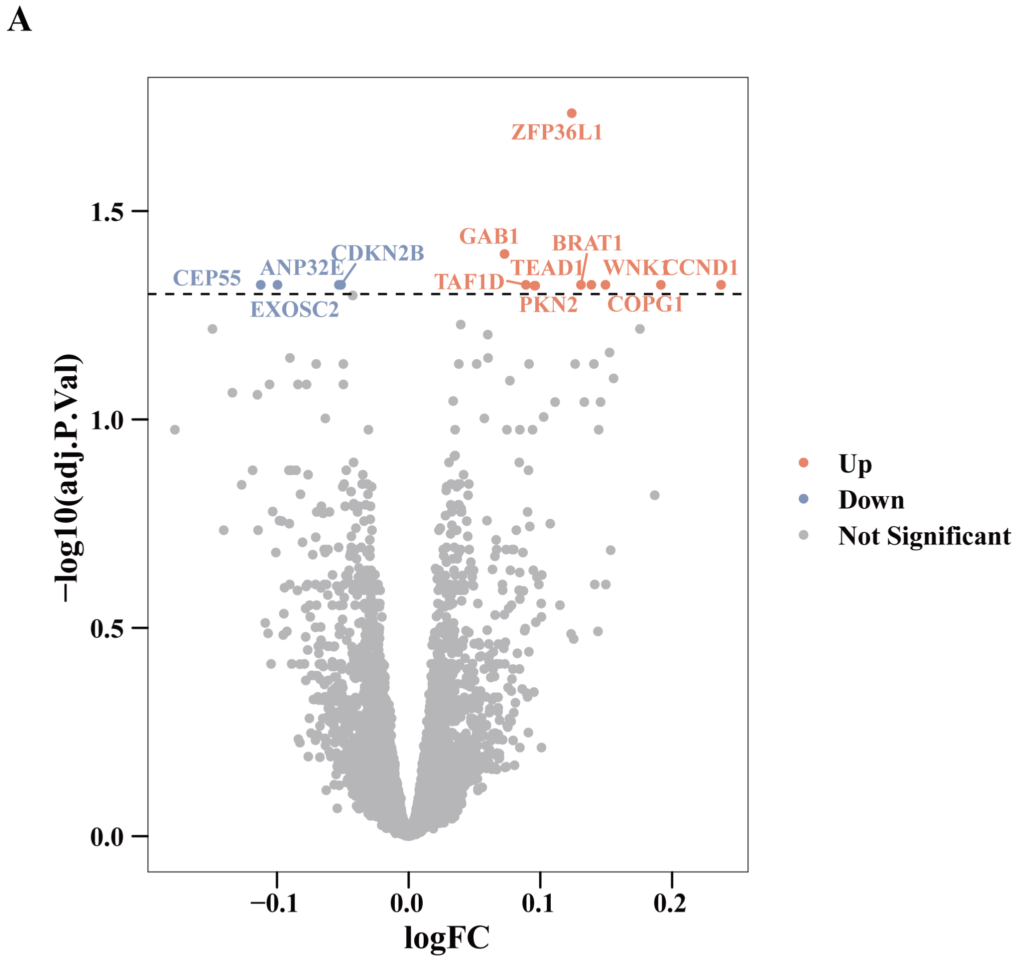


**Supplementary Figure 12. Differences in essentiality of CS-related genes between high and low senescence signature groups in pan-cancer.**

# Supplementary Figure 13

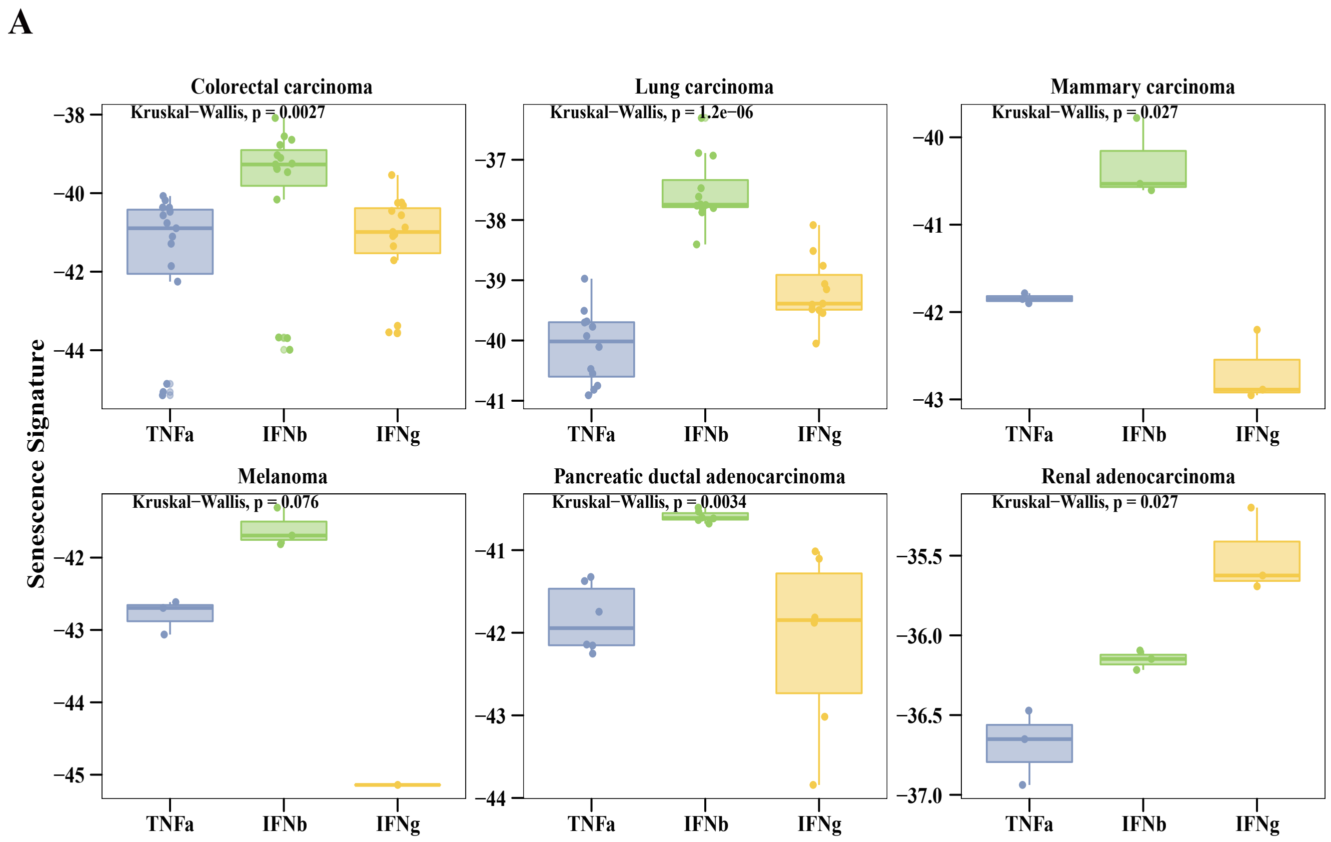


**Supplementary Figure 13. Comparison of 3 immunotherapy (stimulation of INF-β, TNF-α and INF-γ) based on senescence signature score across 6 cancer types from RTM28723893 of TISMO database.**

# Supplementary Figure 14

A

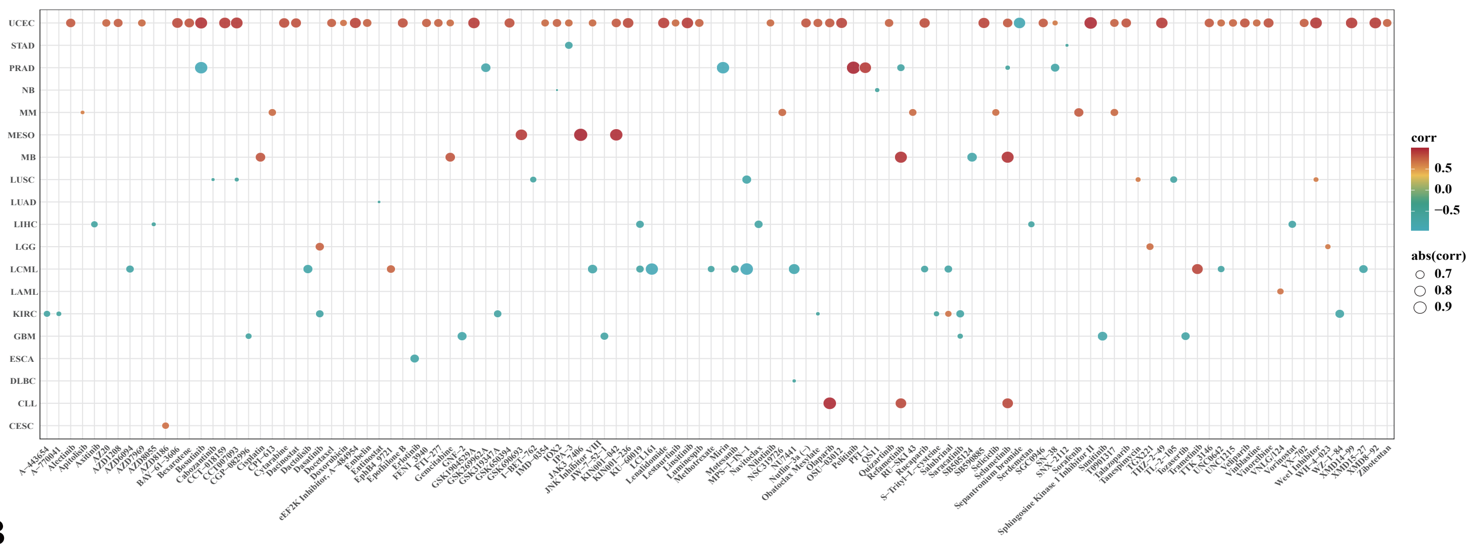


B

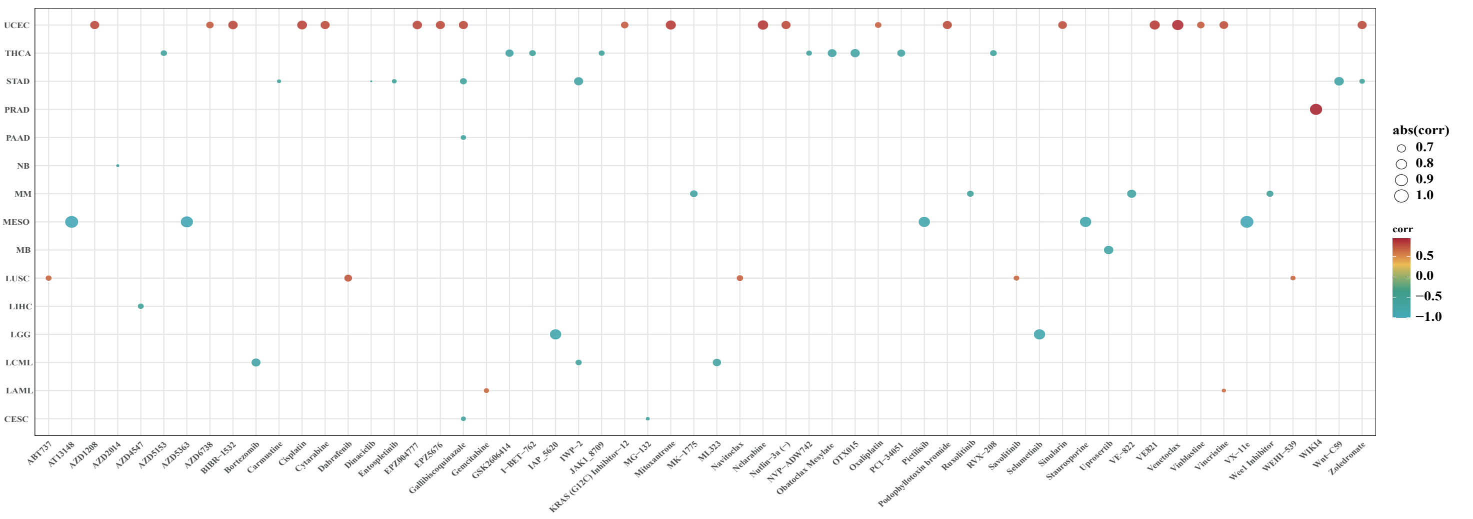


**Supplementary Figure 14. The correlation of senescence signature with drug sensitivity on GDSC1 data (A) and GDSC2 data (B) of pan-cancer (t test, all P < 0.05).**

# Supplementary Figure 15

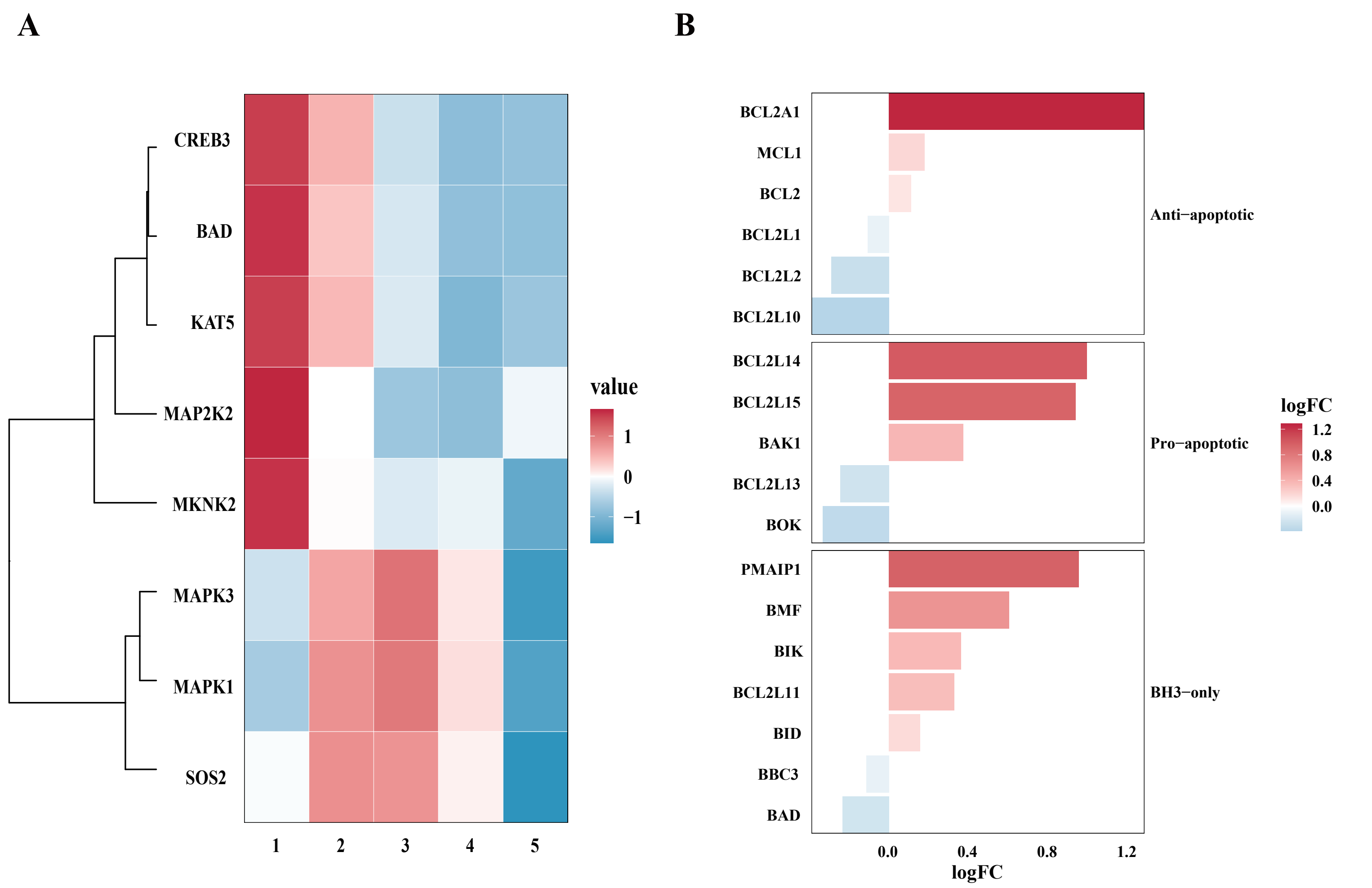


**Supplementary Figure 15. The association of senescence signature with MAPK signaling pathway genes and BCL-2 family genes expression in pan-cancer.**

**(A) MAPK signaling pathway genes expression decreased among CS groups 1-5 of pan-cancer.**

**(B) BCL-2 family genes differential expression between high and low senescence signature groups of pan-cancer.**

# Supplementary Figure 16

A

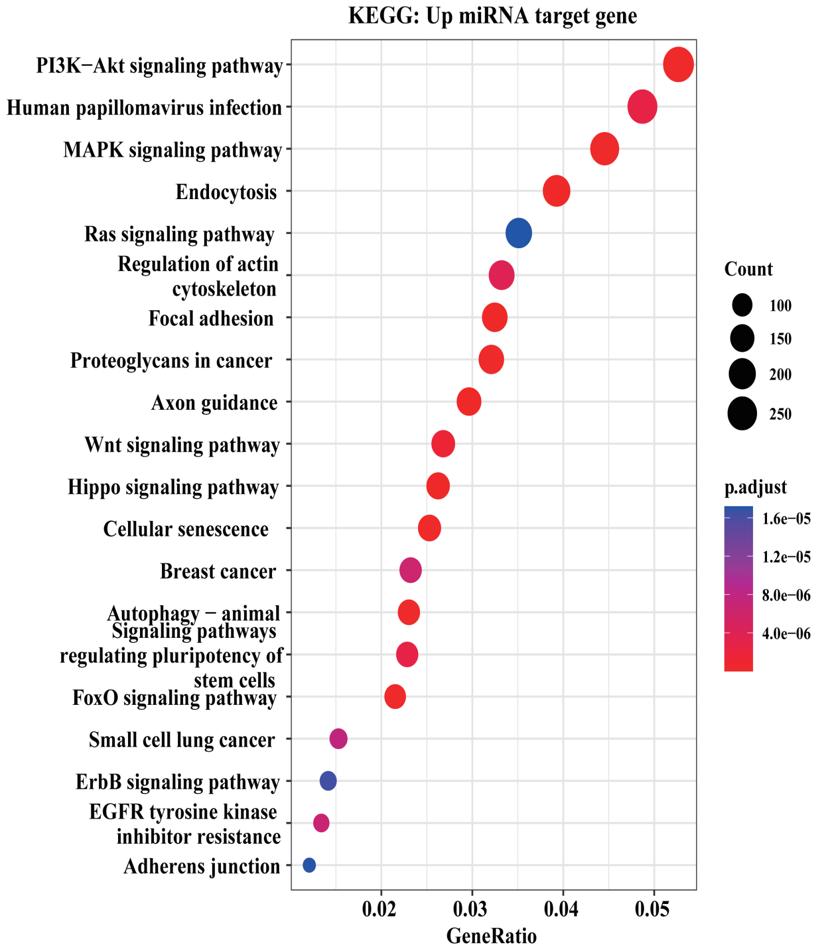


**Supplementary Figure 16. Enriched signal pathways for upregulated expressed miRNAs of high senescence signature group in pan-cancer by KEGG enrichment analysis.**

# Supplementary Figure 17

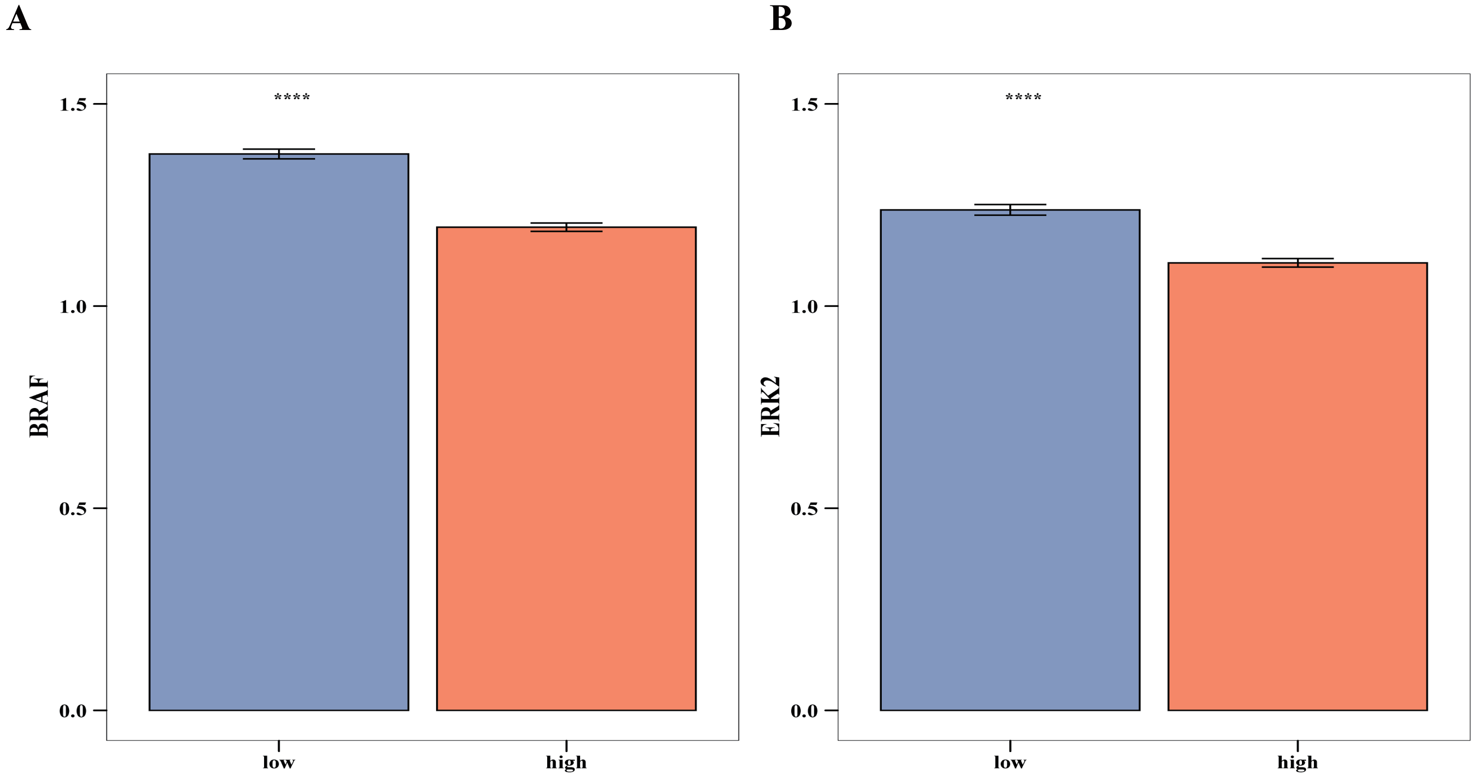


**Supplementary Figure 17. Levels of protein validation of differential expression of MAPK signaling pathway proteins, BRAF (A) and ERK2 (B), between high and low senescence signature groups of pan-cancer.**

# Supplementary Figure 18

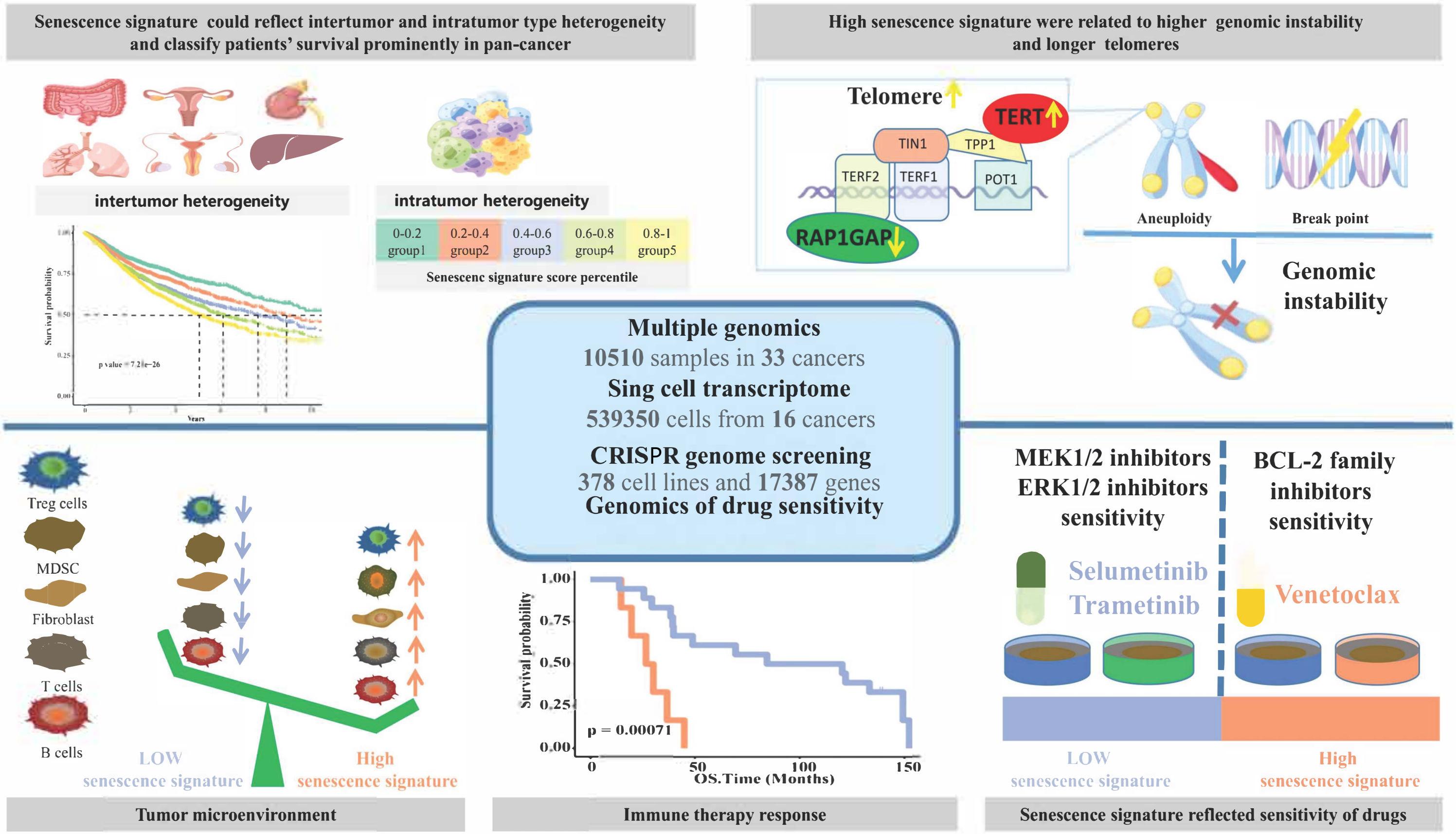


**Supplementary Figure 18. Graphical abstract.**

**We defined an index to quantify CS levels, called "senescence signature". We found that senescence signature effectively reflects intertumor and intratumor type heterogeneity and that patients with higher senescence signature had worse prognosis. Higher senescence signature score was related to higher genomic instability, longer telomeres, higher lymphocytic infiltrate and higher pro-tumor immune infiltrate (Treg cells and MDSCs). Single-cell analysis further revealed malignant cells and immune cells exhibit a consistent evolutionary trend at the CS level , with a higher degree of interaction between malignant cells and immune cells in the high senescence signature group. Senescence signature could be used to predict immune therapy response and drug sensitivity to MEK1/2 inhibitors, ERK1/2 inhibitors, and BCL-2 family inhibitors. (By Figdraw)**
